# Novel Sub-Clustering of Class III Skeletal Malocclusion Phenotypes in a Southern European Population Based on Proportional Measurements

**DOI:** 10.3390/jcm9093048

**Published:** 2020-09-22

**Authors:** Leixuri de Frutos-Valle, Conchita Martín, José Antonio Alarcón, Juan Carlos Palma-Fernández, Ricardo Ortega, Alejandro Iglesias-Linares

**Affiliations:** 1 Section of Orthodontics, Faculty of Odontology, Complutense University, 28040 Madrid, Spain; leixuridefrutos@gmail.com (L.d.F.V.); mariacom@ucm.es or; 2 Section of Orthodontics, Faculty of Odontology, University of Granada, 18071 Granada, Spain; jalarcon@ugr.es; 3 Section of Radiology, Faculty of Odontology, Complutense University, 28040 Madrid, Spain; ricardoortega@odon.ucm.es; 4 BIOCRAN (Craniofacial Biology) Research Group, Complutense University, 28040 Madrid, Spain

**Keywords:** malocclusion, angle Class III, phenotype, principal component analysis, clustering, orthodontics

## Abstract

Current phenotypic characterizations of Class III malocclusion are influenced more by gender or ethnic origin than by raw linear skeletal measurements. The aim of the present research is to develop a Class III skeletal malocclusion sub-phenotype characterization based on proportional cranial measurements using principal component analysis and cluster analysis. Radiometric data from 212 adult subjects (115 women and 96 men) of southern European origin affected by Class III skeletal malocclusion were analyzed. A total of 120 measurements were made, 26 were proportional skeletal measurements, which were used to perform principal component analysis and subsequent cluster analysis. The remaining 94 supplementary measurements were used for a greater description of the identified clusters. Principal component analysis established eight principal components that explained 85.1% of the total variance. The first three principal components explained 51.4% of the variance and described mandibular proportions, anterior facial height proportions, and posterior–anterior cranial proportions. Cluster analysis established four phenotypic subgroups, representing 18.4% (C1), 20.75% (C2), 38.68% (C3), and 22.17% (C4) of the sample. A new sub-clustering of skeletal Class III malocclusions that avoids gender influence is provided. Our results improve clinicians’ resources for Class III malocclusion and could improve the diagnostic and treatment approaches for this malocclusion.

## 1. Introduction

Skeletal Class III malocclusions are among the most challenging malocclusions to treat, first because of the complexity of achieving an optimal treatment outcome [1,2,3] and second, because the clear genetic component determines the prognosis of this type of malocclusion [4,5,6,7,8,9]. Distinguishable ethnic differences have been described in this type of malocclusion, with prevalences ranging from 4.76% [10] to 31.4% [11] in Asian populations to rates below 11% in countries such as Australia (2.5%) [12] Italy (4.3%) [13], Colombia (5.8%) [14], South India (4.1%) [15], Iran (7.8%) [16], and Central Turkey (10.3%) [17]. Apart from absolute prevalence rates, clear indicators of the complexity and importance of this malocclusion are the fact that more than half the patients undergoing orthognathic surgery have skeletal Class III malocclusion and that it carries the highest need-for-correction score according to the index of the need for functional orthognathic treatment (IOFTN) [18,19,20,21,22]. Moreover, varying numbers and Cass III phenotypes are described, depending on the origin of the study population [23,24,25,26,27,28]. The resulting facial characteristics and skeletal structure vary according to the intrinsic features of each ethnic group with skeletal Class III malocclusion [29,30,31,32], which induces notable heterogeneity in the skeletal Class III diagnosis.

With respect to skeletal structure, differences in facial and cranial morphology have been observed between genders. Cranial measurements by gender indicate that raw linear measurements are higher in males than in females, whereas the raw values of these measurements are frequently of lower magnitude in males [29,30,31]. Due to these differences in total linear size, gender-related sub-phenotypic differences have been found in cases of skeletal Class III malocclusion in the same population [28,33,34].

This potential influence of gender and origin suggests that a novel diagnostic method of skeletal Class III malocclusion may be warranted, based exclusively on proportional measurements, and thus avoiding raw linear skeletal measurements. The primary aim of this study, therefore, is to design a simple and manageable clinical sub-phenotypic classification system for skeletal Class III malocclusion that will facilitate future analysis of outcome or prognostic features in Class III subjects. The aim of the present research is to characterize Class III skeletal malocclusion sub-phenotypes on the basis of proportional cranial measurements using principal component analysis and cluster analysis.

## 2. Experimental Section

### 2.1. Study Sample

A sample of 212 subjects (115 women and 96 men) of Southern European origin diagnosed with skeletal Class III malocclusion were selected. All participants were in phases IV or V of the cervical vertebral maturation stage (CVMS) [35] and, therefore, had practically completed their growth. All participants met the following inclusion criteria: molar or canine Class III malocclusion without loss of anterior space, Wits appraisal < −0.5 and/or ANB ≤ 0. The exclusion criteria were the absence of or a low-quality lateral radiograph or individuals who did not sign informed consent. The full sample selection criteria, as well as the sample size estimation, are detailed in a previous study in which the same population was used [33].

The study protocol was approved by the Institutional Ethics Committee (CE) of Complutense University of Madrid (Clinical Research Ethics Committee of the San Carlos Clinical Hospital of Madrid, reference 17/063), safeguarding the rights and interests of the people participating in the research, in accordance with the principles of the Declaration of Helsinki [36].

### 2.2. Measurements/Assessments: Cephalometric Measurement Selection and Analysis

The lateral cephalometric radiographs used for this study were taken as diagnostic records prior to orthodontic treatment between 1995 and 2019. Gender and ethnicity were recorded. Once all lateral radiographs were obtained, the CVMS of each patient was recorded and a single operator (L.F.-V.) imported the radiographs into Dolphin Imaging software (11.0, Dolphin Imaging and Management Solutions, Chatsworth, California) where they were calibrated.

Once calibrated, cephalometric measurements were made incorporating the respective cephalometric points. A total array of 26 proportional skeletal variables were selected for the characterization of Class III patients (Table 1) and used to obtain an *n* number axes model by principal component analysis and subsequent sub-phenotypic classification of clusters. In addition, 94 supplementary cephalometric measurements (7 airway, 15 soft tissue, 25 teeth, and 47 linear and angular skeletal) were used for a supplementary description of the skeletal proportional clusters and analyzed in order to complete the sub-clustering model.

### 2.3. Statistical Analysis

#### Multivariate Analysis

Principal component analysis was performed to summarize and reduce the number of proportional variables used in cephalometric analysis, minimizing any potential loss of information. The amount of information incorporated in each principal component is called variance. The more the information incorporated into a principal component, the greater the variance; and closely correlated original variables require fewer components to explain the variability in the results. Therefore, we sought a model that incorporated a few principal components and could explain a large portion of the total variance with a minimum loss of information. For this purpose, we used Varimax axis rotation with post hoc Kaiser’s standardization.

Subsequently, a mixed cluster analysis (Coheris Analytics SPAD version 9.1) was performed to establish *n’* homogeneous sub-phenotypes of skeletal Class III malocclusion. Ward’s criterion was used with the objective of achieving the lowest dispersion in the identified clusters. Subsequently, a graphical representation [37] of the defined clusters was obtained by generating the cephalometric trace closest to the nucleus of each cluster and adjusting it to the measurements of variables of each group.

The supplementary cephalometric variables, which were recorded simultaneously during cephalometric analysis, were assessed to determine their involvement in the clusters. Finally, by means of the chi-square test, the relationship between CVMS and gender with respect to the generated sub-phenotypes was established.

### 2.4. Method Error

Fifteen lateral cephalometric radiographs were randomly selected from the 212 participants and analysis was replicated by the main operator at a 3 week interval. A Student’s t test for two-tailed paired samples was used to determine the reliability of the cephalometric measurements, establishing a *p*-value greater than 0.05 for all comparisons. The intraclass correlation coefficient of bidirectional mixed effects for absolute agreement (ICC) [38] and the Dahlberg formula [39] were also calculated.

## 3. Results

### 3.1. Method Error

The value of intraclass correlation coefficients was <65% in one instance (63.1% ANS − PNS/Go-Pg (%)) and <80% in two others (73.6% ANS − PNS/SN (%); 71.3% ANS − PNS/Co-A), indicating good reliability. The accuracy of measurements, as determined by the Dahlberg formula, had an error value ranging from 0 (N − ANS/ANS − Me); ANS − PNS/Me-Go) to 15.5 (Pog − NB/A − NPo).

### 3.2. Description of the Main Components

**Figure 1:**
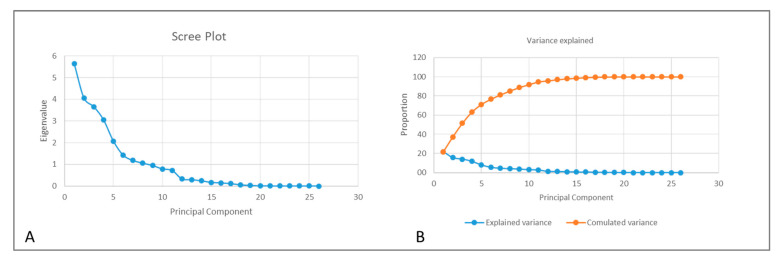
Scree plot of main components illustrating the eigenvalue of each main axis (**A**), the percentage of variance explained by each axis and the cumulative percentage of total variance explained (**B**). The eight main components represented 85.1% of the variance.

Principal component (PC) analysis explained 85.1% of the variance across the 8 principal axes generated in the present Class III sample (Figure 1). The first three principal components primarily described mandibular proportions (SN/GoMe; ANS − PNS/Me − Go; ASN − PNS/Go-Pg), anterior facial proportions (UFH; LFH/TFH; Face Ht Ratio) and anteroposterior cranial proportions (S − Go/N − Me; PFH:AFH), and represent more than 50% of the total variance (51.4%). In all, 21.7% of the variance was represented by the mandibular proportions in PC1: SN/GoMe; ANS − PNS/Me − Go and ASN − PNS/Go − Pg among others. PC2 accounted for 15.6% of the variance and was represented by variables that indicate facial proportions (UFH; LFH/TFH; Face Ht Ratio), while PC3 accounted for 14.1% of the variance and was represented by variables that indicate anteroposterior cranial proportions (S − Go/N − Me; PFH:AFH, Ar − Go/ANS − Me).

PC4 represented 11.7% of the total variance and was composed of proportional variables related primarily to the posterior portion of the face, including S − Ar/Ar − Go, S − Ar/S − Go, Ar − Go/S − Go and S − Ar/N − ANS. PC5 (8% of total variance) only included two variables, both of which relate to proportions of the jaw (ANS − PNS/SN and ANS − PNS/Co − A). PC6 (5.5% of total variance) was primarily composed of variables relating to the occlusal plane. PC7 represented 4.5% of the variance, and contained proportions related to mean facial length (Co − A/Co − GnM; Ar − A/Ar Gn) and the maxillary–mandibular projection (A − N Perp/Pg − N Perp). Finally, PC8 was composed of only two variables (SNB/Articular Angle, Pg − NB/A − NPo), and contributed the least to total variance (4.1%). A detailed description of the 8 main axes is provided in Table 2.

### 3.3. Proportional Sub-Phenotypic Patterns

**Figure 2:**
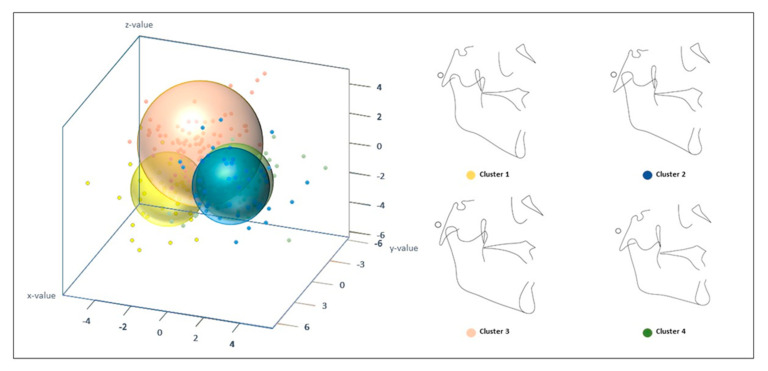
3D representation of the 4 clusters resulting from the cluster analysis and proportional cluster patterns. C1 represented 18.4% of the sample, C2 represented 20.75%, C3 38.68%, and C4 22.17% of the total sample. For the rest of the description see text.

Subsequent cluster analysis defined four homogeneous sub-phenotypes of skeletal Class III malocclusion with clearly identifiable differences between them. A graphical representation of these four established clusters is detailed in Figure 2. The first proportional group (C1) includes 39 subjects, representing 18.4% of the total population. Out of all four sub-phenotypes identified, C1 was the most severe Class III skeletal malocclusion, with the largest maxillomandibular difference and the highest lower facial height. The ratios between the mandibular and maxillary measurements were the lowest among all clusters (Ar − A/Ar − Gn; Co − A/Co − Gn; ANS − PNS/Me − Go; ANS − PNS/Go − Pg). Similarly, the ratio of mandibular measurements to those of the anterior cranial base were also the lowest (SN/GoMe; SN/Go − Pg). Furthermore, individuals in C1 possessed the greatest ratio of lower facial height to total anterior facial height (ANS − Me:N − Me) (Supplementary Table S1 and Figure 2).

The second sub-phenotype (C2) contained 20.75% of the population, with a total of 44 subjects. C2 was characterized by a skeletal Class III malocclusion of maxillary origin with bimaxillary retrusion. This sub-phenotype had decreased angular proportions compared to those of the other groups, as was observed in the relationships between the Sella-Nassion-Point A (SNA) angle and the saddle angle (SN − Ar/SNA) as well as the Sella-Nassion-Point B (SNB) angle to the articular angle (Articular angle/SNB); this a characteristic was also observed in the additional skeletal measurements performed (Supplementary Table S1), indicating a biretrusive pattern. C2 individuals were also characterized by an increased proportion of the posterior cranial base in relation to the mandibular ramus (S − Ar/Ar − Go), while the proportion of the mandibular ramus to posterior facial height (Ar − Go/SGo) and lower anterior facial height (Ar − Go/ANS − Me) were the lowest among all groups. Therefore, C2 individuals possessed the lowest mandibular ramus height of all four groups (Supplementary Table S1). This subgroup also presented with the lowest ratio of posterior facial height to anterior facial height (PFH:AFH; S − Go/N − Me), and the occlusal plane was proportionally more inclined than the mandibular plane with respect to facial height (Occlusal plane to FH/MP − FH) in comparison to the other subgroups (Supplementary Table S1 and Figure 2).

The third proportional group (C3) was composed of the largest number of subjects in the sample (82), representing 38.68% of the sample population. C3 individuals possessed Class III malocclusions with a proportionally smaller mandibular body size compared to the that of rest of the sub-phenotypes, but this is compensated for by the total length of the mandible. Specifically, C3 individuals presented with the lowest ratio of mandibular body length to total mandibular length (Go − Gn/Co − Gn), while the ratio of both the anterior cranial base and maxillary body length to the mandibular body (SN/Go − Pg; SN/GoMe; ANS − PNS/Me − Go; ANS − PNS/Go − Pg) were the largest of the four sub-phenotypes. The C3 subgroup was also characterized by the lowest proportional relationships between the posterior cranial base and both the mandibular ramus (S − Ar/Ar − Go) and posterior facial height (S − Ar/SGo), coinciding with the fact that C3 possessed the highest posterior facial height and ramus height when compared with those of the other subgroups (Supplementary Table S1). As a result, a proportionally higher posterior facial height with respect to the anterior facial height (PFH:AFH) was observed in this subgroup (Supplementary Table S1 and Figure 2).

Lastly, 22.17% of the total sample population was classified as sub-phenotype C4, with a total of 47 subjects. C4 individuals presented with the least severe skeletal Class III malocclusion of the four sub-phenotypes, with a mandibular component, a decreased anterior facial height, and a reduced mandibular plane. C4 was characterized by the highest proportional value of the total maxillary length with respect to full mandibular length (Ar − A/Ar − Gn; Co − A/Co − Gn). In turn, the ratio of the mandibular body length to full mandibular length (Go − Gn/Co − Gn) was the highest of all groups. C4 individuals also possessed the lowest ratio of lower facial height to anterior facial height (ANS − Me:N − Me) and the highest ratio of the middle third facial height to both the anterior facial height (N − ANS/N − ANS + ANS − Me) and to the lower facial height (N − ANS/ANS − Me). As a result, C4 subjects have proportionally reduced heights in the lower third of anterior portions of the face. Furthermore, these individuals also possess the highest proportional value between posterior face height and anterior face height (S − Go/N − Me; PFH:AFH) due to the low anterior face height of C4 compared to that of the other three sub-phenotypes (Supplementary Table S1 and Figure 2).

### 3.4. Description of the Supplementary Variables in Each Proportional Sub-Phenotypic Cluster

For the supplementary skeletal variables, the equivalence of linear and angular measurements with respect to proportional measurements were analyzed by sub-phenotype. Measurements of soft tissue facial height corresponded to those of skeletal facial height. The longest lower third of the face (Sn’-Me’) was observed in C1 and the shortest in C4. Likewise, C1 presented with the lowest retrusion of the lower lip and C4 the highest, while the highest inclination and protrusion of the lower incisor with respect to A-Pg was presented by C1 and the lowest by C4. With respect to the airway, C2 had the shortest upper airway, while C3 had the widest upper airway.

Finally, with respect to the supplementary descriptive dental variables, C1 presented with the lowest overbite and overjet, while the greatest were observed in C4. C2 was characterized by the lowest angulation of the upper incisor with respect to the Nassion-Point A (NA) plane, the Sella-Sella-Nassion (SN) plane, the palatal plane, and the Franckfort Horizontal (FH) plane, while C4 presented the highest angulations of the upper incisor with respect to the same planes. (A more detailed description of the supplementary variables that describe each cluster can be found in Supplementary Table S1).

### 3.5. Gender and Maturation Stage Distribution in Each Proportional Sub-Phenotypic Pattern

Regarding gender and CVMS, their distribution was homogenous in all of the four clusters generated (*p* > 0.05).

## 4. Discussion

Cluster analysis for the phenotypic classification of skeletal Class III malocclusion is being increasingly used to establish specific sub-phenotypes within the large variations of this malocclusion [23,24,25,26,27,34,40,41]. The establishment of appropriate, specific, distinguishable, and easy-to-use clinical sub-phenotypes would facilitate future studies focused on treatment outcomes, relapse prognosis, or even diagnostic characterization of this particular malocclusion, which may ultimately lead to more appropriate therapeutic protocols for each sub-phenotype.

In this study, 212 lateral cephalometric radiographs of subjects with skeletal Class III malocclusion were analyzed. Twenty-six skeletal proportional variables were used to perform principal component analysis and subsequent cluster analysis. Eight main axes were obtained that represented 85.1% of the total variance, allowing for subsequent cluster analysis which generated two possible configurations of four or six clusters. The six-cluster model was discarded as three of these clusters were highly similar, with no identifiable differences being apparent during graphical recreation. Therefore, the six-cluster model would complicate the clinical diagnosis of this type of malocclusion, contrary to the main objective of this type of analysis. In comparison with other well-designed studies that use skeletal, dental, soft tissue, and some proportional variables for multivariate analysis, in this study, only proportional variables were used to construct the final Class III sub-phenotypes. This method allows us to obtain clinically simplified clusters unaffected by the differences in raw values, which could be affected by gender or other factors. Several studies have demonstrated the differences in anterior facial height and other variables between men and women as well as between ethnic groups [28,29,30,31,32]. Linear measurements in men are higher than in women, with greater mandibular size and facial height. Zacharopoulos et al. [30] conducted a study with the aim of providing an anthropometric facial profile in a Greek population. They observed statistically significant differences in the head and face region between genders, and when compared against published data on North American Caucasians, statistically significant differences were observed. Similarly, Celebi et al. [31] analyzed the sexual dimorphism of facial features in Italian and Egyptian populations and observed that some features, including total facial height, upper facial height, lower facial height, and mandibular measurements were significantly greater in both Italian and Egyptian men than in women. In addition, they also found significant differences in facial morphology between the two sample populations. Due to these existing differences, some generated clusters have been composed mainly or entirely of males or females. Moreover, in a previous study [33] we concluded that in 66.6% of the clusters generated, a gender effect was involved. Specifically, two clusters from the previous study were composed mainly of males (85.7% and 79.4%), while two others were composed mainly or entirely of women (73.4% and 100%). Few studies indicate the total number of each gender in the different subgroups identified. Nevertheless, differences in gender within clusters can be observed in other studies; for instance, in Li et al. [34], two of the four characterized clusters were disproportionately made up of females (67.4% and 75%).

In the present study, the most severe sub-phenotype of skeletal Class III malocclusion was observed in C1, which accounted for the smallest proportion of the sample population (18.4%) among all subgroups, similar to the case in previous studies [26,33,34,41]. However, in this case, gender distribution was proportional. C1 showed a Class III malocclusion of mixed maxillary–mandibular origin [42], with the largest maxillary–mandibular difference, the smallest maxillary size, and a relatively high mandibular size of all four clusters. The highest anterior facial height was also observed in C1, but the mandibular plane was normal. The low frequency of severe Class III malocclusion observed here and in several previous studies may be due to the selection process: the selection of an ANB <1 or 0 [26,33,34,41] and a Wits appraisal <0 or −2 mm [26,33,34] may allow for the inclusion of less severe skeletal Class III malocclusion subjects.

The third type of skeletal Class III phenotype (C3) contained the highest percentage of the sample population (38.68%) and represented a skeletal Class III malocclusion with a proportionally increased mandibular ramus, a proportionally decreased mandibular body, and a greater total mandibular size. The C3 cluster represented a skeletal Class III malocclusion of mixed origin and a mesofacial pattern with a decreased mandibular size compared to the rest of the clusters. Despite possessing the greatest maxillary length of all subgroups, C3 still possesses a mesofacial pattern and a slight maxillary retrusion. These characteristics are similar to what was observed in clusters that represented a large portion of the sample in previous studies [26,34,41] that confirm skeletal Class III of mixed origin as the most frequent type of skeletal Class III malocclusion [42].

The second most representative cluster in this study, C4 (22.17%), was characterized by less severe skeletal malocclusion of mandibular origin, with the smallest maxillo-mandibular difference and a diminished anterior facial height and mandibular plane. Similar characteristics were described in sub-phenotypes found in previous studies that showed a mild skeletal Class III malocclusion of mixed maxillary–mandibular origin, with a flat mandibular plane [26,33].

Finally, C2 represented 20.75% of the total sample population and was characterized by a malocclusion of maxillary origin, a proportionally smaller mandibular ramus, a posterior facial height proportionally lower than the anterior facial height, and a more retracted maxillary–mandibular position. When compared with the supplementary skeletal measurements, C2 demonstrated the lowest SNA and SNB angles of all subgroups. This type of Class III has been described in previous studies, also representing the second most frequent type of Class III malocclusion (19.5%) [42]. Similar characteristics were found in other studies, where the clusters representing a skeletal Class III malocclusion of maxillary origin were the second most representative clusters [33] or even the most representative [26,41]. Despite the observation of a skeletal Class III malocclusion of maxillary origin in previous studies, the sub-phenotype found in the present study (C2) has not been previously characterized [34]. This could be due to the different ethnic origin of the populations [28]; a skeletal malocclusion of maxillary origin is described in Caucasian samples [26,33] or in samples with a greater Caucasian component [41] but not in Asian samples [7].

This configuration of four clusters is comparable to that found in previous studies. However, this type of analysis has led to models with 3 [24,27,40], 4 [34], 5 [26,41], 6 [33], 7 [23], and 14 [25] clusters. The variations in the number of clusters identified may be due to differences in the type of cluster analysis employed, whether hierarchical [24] or diffuse [27,40], in comparison with the mixed principal component analyses used in our study. The reduction to four clusters found may also be a result of the use of proportional variables for multivariate analyses instead of linear, angular, and proportional skeletal variables; using proportional variables enabled us to avoid gender-dependent effects on clustering, thus reducing the number of clinical clusters. The large number of subgroups found in other studies [23,25] could be due, among other reasons, to the lack of principal component analysis prior to cluster analysis or to the inclusion of dental Class III malocclusion subjects, thus including subgroups with Class III dental characteristics in addition to Class III skeletal features [25].

The supplementary variables obtained in this study complement the proportional variables described above. The soft tissue variables correlated to the corresponding skeletal patterns, for example, the cluster that had the proportionally greatest lower facial height (C1) also possessed the greatest soft tissue proportions, as was true for the cluster that presented with the lowest facial height (C4). On the other hand, the position of the lower lip with respect to the S Line and E-Plane was more related to the inclination and position of the lower incisor with respect to the A-Pg plane. We observed that the lowest retrusion of the lower lip in C1 coincided with the highest inclination and protrusion of the lower incisor with respect to the A-Pg plane, while the highest retrusion of the lower lip in C4 coincided with the lowest inclination and protrusion of the lower incisor (see Supplementary Table S1). These results are consistent with multiple studies that have established that the labial position depends fundamentally on the position and inclination of the lower incisors [43,44], while the soft tissues closest to their respective skeletal parts follow a pattern closer to them [45].

The use of principal component analysis established a total of eight principal axes, which were used in subsequent cluster analysis. The eight principal components obtained in this study accounted for 85.1% of the total variance, higher than that observed in other studies, where the total variance ranged from 67% [41] to 81.2% [26] with a number of principal components ranging from five [41] to six [26,34]. The higher percentage of total variance accounted for by the current model may be due to the use of a set of variables focused on the proportional skeleton. Despite the use of a smaller group of variables for cluster analysis, the first three principal components accounted for 51.4% of the total variance, results that are in agreement with previous studies [26,34,41]. The use of proportional variables to obtain the principal components makes it difficult to compare with other studies. In spite of this, similarities with other studies can be observed in the first principal components, since they described sagittal and vertical variables [26,34,41]. In our study, PC1 describe mandibular proportions, i.e., it represents the relationship of the mandible with respect to the cranial and maxillary base among others (SN/GoMe; ANS − PNS/Me − Go; ASN − PNS/Go − Pg); this representation is similar to PC1 of previous articles describing sagittal parameters [26,41]. PC2 and PC3 describe proportions related to anterior facial height (UFH; LFH/TFH; Face Ht Ratio) and proportions related to posterior–anterior height (S − Go/N − Me; PFH:AFH, Ar − Go/ANS − Me), respectively, and their results are comparable with those of previous articles where vertical parameters are described in PC2 [33,34] or vertical and sagittal parameters in PC3 [33,41]. In previous studies that used skeletal, dental, and soft tissue variables to obtain principal components, parameters indicating the position of the lower incisor were part of principal components, (PC2 [41], PC3 [26,34]) and explained a high percentage of variance (14.66% [41], 13.25% [26], 12.16% [34]). The differences between our study and these previous works are primarily due to the use of skeletal proportional variables instead of skeletal, dental, and soft tissue variables. This type of analysis may aid future genetic studies, since an increasing number of studies are performing first principal component analysis to relate principal components to genetics [6,46]. Genes involved in vertical craniofacial discrepancies and others involved in horizontal discrepancies have been identified, as well as those involved in the mandibular prognathism observed in different ethnicities [6,7,47].

The differences found between ethnicities both in the phenotypic sub-classification of skeletal Class III malocclusions [28], as well as in the genes involved in mandibular prognathism [6,7,47], show the need for an appropriate classification of this malocclusion in different ethnicities to facilitate future studies on prognosis and treatment outcomes. The present diagnostic tool might modify, or at least classify, current treatment plan strategies based on skeletal pattern. This method might be useful to analyze how these sub-phenotypes respond to different orthopedic and orthodontic Class III treatment strategies based on the results of future randomized clinical trials aimed at analyzing the effects of any Class III therapy. To achieve this, precise but feasible skeletal Class III classifications may help configure Class III subtypes that provide a clear and simple diagnostic tool without being affected by gender- or ethnicity-dependent effects.

## 5. Conclusions

The classification of skeletal Class III malocclusion by cluster analysis after a principal component analysis based on proportional variables, provides a clear, concise sub-phenotypic classification and avoids potential gender-dependent effects that can occur when raw values are used. This classification is more clinically useful and may facilitate diagnoses, as well as improving future studies on treatment outcomes, prognoses, or even diagnoses of this malocclusion.

## Figures and Tables

**Table 1:** Skeletal proportional craniometric variables used in PCA.

P-A Face Height (S-Go/N-Me) (%)	Anterior Cranial Base (SN)/Length of Mand Base (Go-Pg) (%)
PFH:AFH (%)	Maxillary length (ANS-PNS)/Anterior Cranial Base (SN) (%)
S-Ar/Ar-Go (%)	Maxillary length (ANS-PNS)/Midface Length (Co-A) (%)
UFH (N-ANS/(N-ANS+ANS-Me)) (%)	Maxillary length (ANS-PNS)/Length of Mand Base (Go-Pg) (%)
LFH/TFH (ANS-Me:N-Me) (%)	Midface Length (Co-A)/Mandibular length (Co-Gn) (%)
Face Ht Ratio (N-ANS/ANS-Me) (%)	Mandibular Body Length (Go-Gn)/Mandibular length (Co-Gn) (%)
SN/GoMe (%)	Ar - A/Ar - Gn (%)
ANS-PNS/Me-Go (%)	Posterior Cranial Base (S-Ar)/Posterior Face Height (SGo) (%)
Articular Angle/SNB (%)	Ramus Height (Ar-Go)/Posterior Face Height (SGo) (%)
Saddle-Sella Angle (SN-Ar)/SNA (%)	Posterior Cranial Base (S-Ar)/Upper Face Height (N-ANS) (%)
Occ Plane to FH/FMA (MP-FH) (%)	Ramus Height (Ar-Go)/Lower Face Height (ANS-Me) (%)
Occ Plane to SN/SN - GoGn (%)	Maxillary Skeletal (A-N Perp)/Mand. Skeletal (Pg-Na Perp) (%)
Cranio-Mx Base (SN-Palatal Plane)/SN - GoGn (%)	Convexity (A-NPg)/Pg - NB (%)

**Table 2:** Sumary of the principal components analysis.

Principal Component	PC 1	PC 2	PC 3	PC 4	PC 5	PC 6	PC 7	PC 8
% of explained variance (a)	21.7	15.6	14.1	11.7	8.0	5.5	4.5	4.1
Cumulated % of explained variance (b)	21.7	37.3	51.4	63.1	71.1	76.5	81.8	85.1
Cephalometric variables (c)	SN/GoMe (%)	UFH (N-ANS/(N-ANS+ANS-Me)) (%)	P-A Face Height (S-Go/N-Me) (%)	S-Ar/Ar-Go (%)	Maxillary length (ANS-PNS)/Anterior Cranial Base (SN) (%)	SNA/Saddle-Sella Angle (SN-Ar) (%)	Midface Length (Co-A)/Mandibular length (Co-Gn)(%)	SNB/Articular Angle (%)
	ANS-PNS/Me-Go (%)	LFH/TFH (ANS-Me:N-Me) (%)	PFH:AFH (%)	Posterior Cranial Base (S-Ar)/Posterior Face Height (SGo) (%)	Maxillary length (ANS-PNS)/Midface Length (Co-A) (%)	Occ Plane to FH/FMA (MP-FH) (%)	Ar - A/Ar - Gn (%)	Pog - NB/Convexity (A-NPo) (%)
	Anterior Cranial Base (SN)/Length of Mand Base (Go-Pg) (%)	Face Ht Ratio (N-ANS/ANS-Me) (%)	Ramus Height (Ar-Go)/Lower Face Height (ANS-Me) (%)	Ramus Height (Ar-Go)/Posterior Face Height (SGo) (%)		Occ Plane to SN/SN – GoGn (%)	Maxillary Skeletal (A-N Perp)/Mand. Skeletal (Pg-N Perp) (%)	
	Maxillary length (ANS-PNS)/Length of Mand Base (Go-Pg) (%)	Cranio-Mx Base (SN-Palatal Plane)/SN – GoGn (%)		Posterior Cranial Base (S-Ar)/Upper Face Height (N-ANS) (%)				
	Mandibular Body Length (Go-Gn)/Mandibular length (Co-Gn) (%)							

(a) shows the variance explained by each principal component; (b) cumulative variance explained by each added principal component; (c) variables with the highest contribution in each PC.

## Supplementary Materials

The following are available online at https://www.mdpi.com/2077-0383/9/9/3048/s1, Table S1: Mean values of proportional skeletal variables and supplementary variables, Table S2: Explanation of cephalometric measurements and abbreviations.
