# Supplementary material for: Novel Sub-Clustering of Class III Skeletal Malocclusion Phenotypes in a Southern European Population Based on Proportional Measurements
Source: J Clin Med. 2020 Sep 22;9(9):3048. doi: 10.3390/jcm9093048 (PMC7565379; doi:10.3390/jcm9093048)
Supplement: Supplementary file 1 [file jcm-09-03048-s001.zip › Supplementary Table 1. Mean values of proportional skeletal variables and supplementary variables. .pptx]

## Slide 1
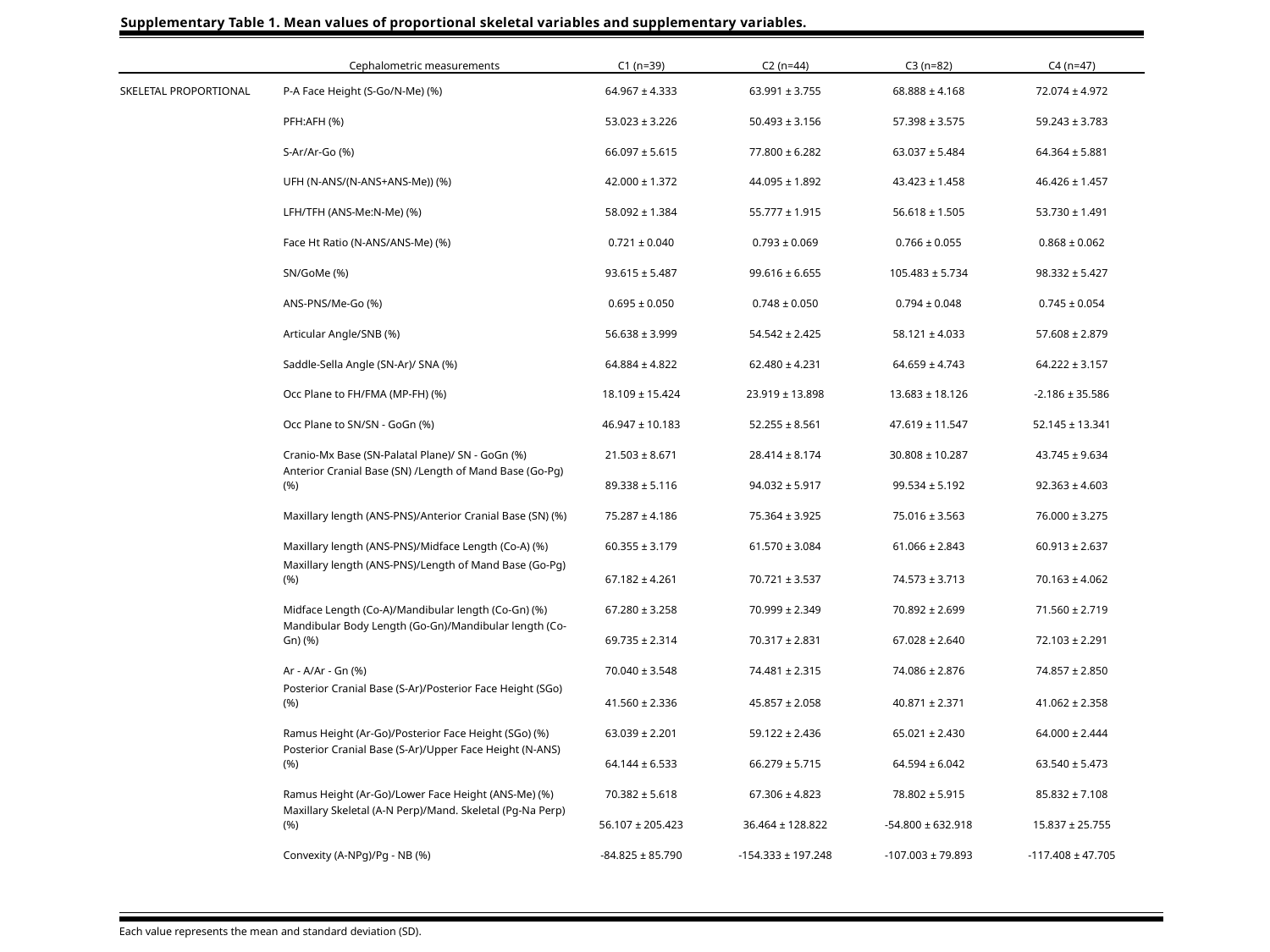

Supplementary Table 1. Mean values of proportional skeletal variables and supplementary variables.
| | Cephalometric measurements | C1 (n=39) | C2 (n=44) | C3 (n=82) | C4 (n=47) |
| --- | --- | --- | --- | --- | --- |
| SKELETAL PROPORTIONAL | P-A Face Height (S-Go/N-Me) (%) | 64.967 ± 4.333 | 63.991 ± 3.755 | 68.888 ± 4.168 | 72.074 ± 4.972 |
| | PFH:AFH (%) | 53.023 ± 3.226 | 50.493 ± 3.156 | 57.398 ± 3.575 | 59.243 ± 3.783 |
| | S-Ar/Ar-Go (%) | 66.097 ± 5.615 | 77.800 ± 6.282 | 63.037 ± 5.484 | 64.364 ± 5.881 |
| | UFH (N-ANS/(N-ANS+ANS-Me)) (%) | 42.000 ± 1.372 | 44.095 ± 1.892 | 43.423 ± 1.458 | 46.426 ± 1.457 |
| | LFH/TFH (ANS-Me:N-Me) (%) | 58.092 ± 1.384 | 55.777 ± 1.915 | 56.618 ± 1.505 | 53.730 ± 1.491 |
| | Face Ht Ratio (N-ANS/ANS-Me) (%) | 0.721 ± 0.040 | 0.793 ± 0.069 | 0.766 ± 0.055 | 0.868 ± 0.062 |
| | SN/GoMe (%) | 93.615 ± 5.487 | 99.616 ± 6.655 | 105.483 ± 5.734 | 98.332 ± 5.427 |
| | ANS-PNS/Me-Go (%) | 0.695 ± 0.050 | 0.748 ± 0.050 | 0.794 ± 0.048 | 0.745 ± 0.054 |
| | Articular Angle/SNB (%) | 56.638 ± 3.999 | 54.542 ± 2.425 | 58.121 ± 4.033 | 57.608 ± 2.879 |
| | Saddle-Sella Angle (SN-Ar)/ SNA (%) | 64.884 ± 4.822 | 62.480 ± 4.231 | 64.659 ± 4.743 | 64.222 ± 3.157 |
| | Occ Plane to FH/FMA (MP-FH) (%) | 18.109 ± 15.424 | 23.919 ± 13.898 | 13.683 ± 18.126 | -2.186 ± 35.586 |
| | Occ Plane to SN/SN - GoGn (%) | 46.947 ± 10.183 | 52.255 ± 8.561 | 47.619 ± 11.547 | 52.145 ± 13.341 |
| | Cranio-Mx Base (SN-Palatal Plane)/ SN - GoGn (%) | 21.503 ± 8.671 | 28.414 ± 8.174 | 30.808 ± 10.287 | 43.745 ± 9.634 |
| | Anterior Cranial Base (SN) /Length of Mand Base (Go-Pg) (%) | 89.338 ± 5.116 | 94.032 ± 5.917 | 99.534 ± 5.192 | 92.363 ± 4.603 |
| | Maxillary length (ANS-PNS)/Anterior Cranial Base (SN) (%) | 75.287 ± 4.186 | 75.364 ± 3.925 | 75.016 ± 3.563 | 76.000 ± 3.275 |
| | Maxillary length (ANS-PNS)/Midface Length (Co-A) (%) | 60.355 ± 3.179 | 61.570 ± 3.084 | 61.066 ± 2.843 | 60.913 ± 2.637 |
| | Maxillary length (ANS-PNS)/Length of Mand Base (Go-Pg) (%) | 67.182 ± 4.261 | 70.721 ± 3.537 | 74.573 ± 3.713 | 70.163 ± 4.062 |
| | Midface Length (Co-A)/Mandibular length (Co-Gn) (%) | 67.280 ± 3.258 | 70.999 ± 2.349 | 70.892 ± 2.699 | 71.560 ± 2.719 |
| | Mandibular Body Length (Go-Gn)/Mandibular length (Co-Gn) (%) | 69.735 ± 2.314 | 70.317 ± 2.831 | 67.028 ± 2.640 | 72.103 ± 2.291 |
| | Ar - A/Ar - Gn (%) | 70.040 ± 3.548 | 74.481 ± 2.315 | 74.086 ± 2.876 | 74.857 ± 2.850 |
| | Posterior Cranial Base (S-Ar)/Posterior Face Height (SGo) (%) | 41.560 ± 2.336 | 45.857 ± 2.058 | 40.871 ± 2.371 | 41.062 ± 2.358 |
| | Ramus Height (Ar-Go)/Posterior Face Height (SGo) (%) | 63.039 ± 2.201 | 59.122 ± 2.436 | 65.021 ± 2.430 | 64.000 ± 2.444 |
| | Posterior Cranial Base (S-Ar)/Upper Face Height (N-ANS) (%) | 64.144 ± 6.533 | 66.279 ± 5.715 | 64.594 ± 6.042 | 63.540 ± 5.473 |
| | Ramus Height (Ar-Go)/Lower Face Height (ANS-Me) (%) | 70.382 ± 5.618 | 67.306 ± 4.823 | 78.802 ± 5.915 | 85.832 ± 7.108 |
| | Maxillary Skeletal (A-N Perp)/Mand. Skeletal (Pg-Na Perp) (%) | 56.107 ± 205.423 | 36.464 ± 128.822 | -54.800 ± 632.918 | 15.837 ± 25.755 |
| | Convexity (A-NPg)/Pg - NB (%) | -84.825 ± 85.790 | -154.333 ± 197.248 | -107.003 ± 79.893 | -117.408 ± 47.705 |
Each value represents the mean and standard deviation (SD).

## Slide 2
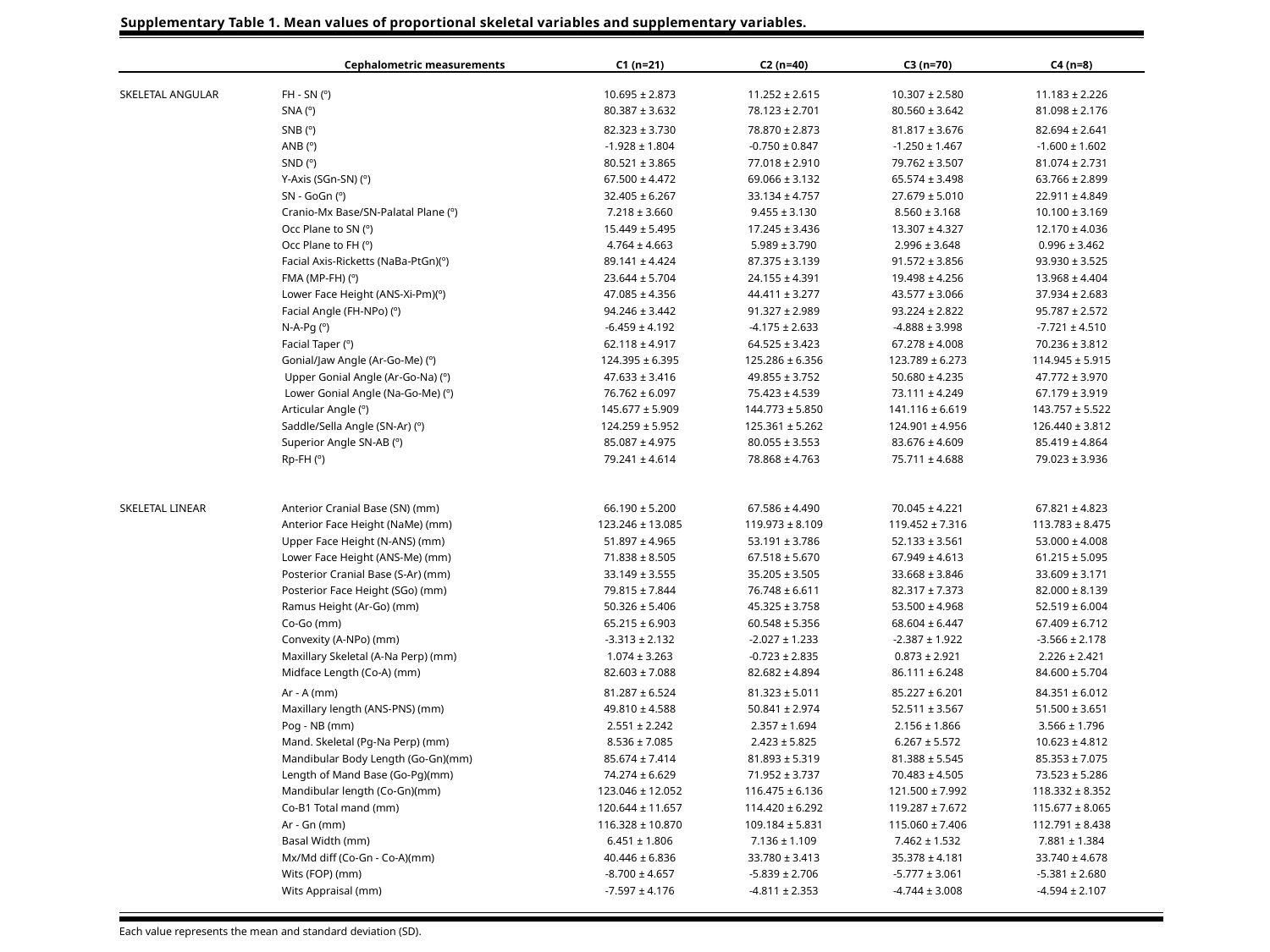

Supplementary Table 1. Mean values of proportional skeletal variables and supplementary variables.
| | Cephalometric measurements | C1 (n=21) | | C2 (n=40) | | C3 (n=70) | | C4 (n=8) | |
| --- | --- | --- | --- | --- | --- | --- | --- | --- | --- |
| SKELETAL ANGULAR | FH - SN (º) | 10.695 ± 2.873 | | 11.252 ± 2.615 | | 10.307 ± 2.580 | | 11.183 ± 2.226 | |
| | SNA (º) | 80.387 ± 3.632 | | 78.123 ± 2.701 | | 80.560 ± 3.642 | | 81.098 ± 2.176 | |
| | SNB (º) | 82.323 ± 3.730 | | 78.870 ± 2.873 | | 81.817 ± 3.676 | | 82.694 ± 2.641 | |
| | ANB (º) | -1.928 ± 1.804 | | -0.750 ± 0.847 | | -1.250 ± 1.467 | | -1.600 ± 1.602 | |
| | SND (º) | 80.521 ± 3.865 | | 77.018 ± 2.910 | | 79.762 ± 3.507 | | 81.074 ± 2.731 | |
| | Y-Axis (SGn-SN) (º) | 67.500 ± 4.472 | | 69.066 ± 3.132 | | 65.574 ± 3.498 | | 63.766 ± 2.899 | |
| | SN - GoGn (º) | 32.405 ± 6.267 | | 33.134 ± 4.757 | | 27.679 ± 5.010 | | 22.911 ± 4.849 | |
| | Cranio-Mx Base/SN-Palatal Plane (º) | 7.218 ± 3.660 | | 9.455 ± 3.130 | | 8.560 ± 3.168 | | 10.100 ± 3.169 | |
| | Occ Plane to SN (º) | 15.449 ± 5.495 | | 17.245 ± 3.436 | | 13.307 ± 4.327 | | 12.170 ± 4.036 | |
| | Occ Plane to FH (º) | 4.764 ± 4.663 | | 5.989 ± 3.790 | | 2.996 ± 3.648 | | 0.996 ± 3.462 | |
| | Facial Axis-Ricketts (NaBa-PtGn)(º) | 89.141 ± 4.424 | | 87.375 ± 3.139 | | 91.572 ± 3.856 | | 93.930 ± 3.525 | |
| | FMA (MP-FH) (º) | 23.644 ± 5.704 | | 24.155 ± 4.391 | | 19.498 ± 4.256 | | 13.968 ± 4.404 | |
| | Lower Face Height (ANS-Xi-Pm)(º) | 47.085 ± 4.356 | | 44.411 ± 3.277 | | 43.577 ± 3.066 | | 37.934 ± 2.683 | |
| | Facial Angle (FH-NPo) (º) | 94.246 ± 3.442 | | 91.327 ± 2.989 | | 93.224 ± 2.822 | | 95.787 ± 2.572 | |
| | N-A-Pg (º) | -6.459 ± 4.192 | | -4.175 ± 2.633 | | -4.888 ± 3.998 | | -7.721 ± 4.510 | |
| | Facial Taper (º) | 62.118 ± 4.917 | | 64.525 ± 3.423 | | 67.278 ± 4.008 | | 70.236 ± 3.812 | |
| | Gonial/Jaw Angle (Ar-Go-Me) (º) | 124.395 ± 6.395 | | 125.286 ± 6.356 | | 123.789 ± 6.273 | | 114.945 ± 5.915 | |
| | Upper Gonial Angle (Ar-Go-Na) (º) | 47.633 ± 3.416 | | 49.855 ± 3.752 | | 50.680 ± 4.235 | | 47.772 ± 3.970 | |
| | Lower Gonial Angle (Na-Go-Me) (º) | 76.762 ± 6.097 | | 75.423 ± 4.539 | | 73.111 ± 4.249 | | 67.179 ± 3.919 | |
| | Articular Angle (º) | 145.677 ± 5.909 | | 144.773 ± 5.850 | | 141.116 ± 6.619 | | 143.757 ± 5.522 | |
| | Saddle/Sella Angle (SN-Ar) (º) | 124.259 ± 5.952 | | 125.361 ± 5.262 | | 124.901 ± 4.956 | | 126.440 ± 3.812 | |
| | Superior Angle SN-AB (º) | 85.087 ± 4.975 | | 80.055 ± 3.553 | | 83.676 ± 4.609 | | 85.419 ± 4.864 | |
| | Rp-FH (º) | 79.241 ± 4.614 | | 78.868 ± 4.763 | | 75.711 ± 4.688 | | 79.023 ± 3.936 | |
| | | | | | | | | | |
| | | | | | | | | | |
| SKELETAL LINEAR | Anterior Cranial Base (SN) (mm) | 66.190 ± 5.200 | | 67.586 ± 4.490 | | 70.045 ± 4.221 | | 67.821 ± 4.823 | |
| | Anterior Face Height (NaMe) (mm) | 123.246 ± 13.085 | | 119.973 ± 8.109 | | 119.452 ± 7.316 | | 113.783 ± 8.475 | |
| | Upper Face Height (N-ANS) (mm) | 51.897 ± 4.965 | | 53.191 ± 3.786 | | 52.133 ± 3.561 | | 53.000 ± 4.008 | |
| | Lower Face Height (ANS-Me) (mm) | 71.838 ± 8.505 | | 67.518 ± 5.670 | | 67.949 ± 4.613 | | 61.215 ± 5.095 | |
| | Posterior Cranial Base (S-Ar) (mm) | 33.149 ± 3.555 | | 35.205 ± 3.505 | | 33.668 ± 3.846 | | 33.609 ± 3.171 | |
| | Posterior Face Height (SGo) (mm) | 79.815 ± 7.844 | | 76.748 ± 6.611 | | 82.317 ± 7.373 | | 82.000 ± 8.139 | |
| | Ramus Height (Ar-Go) (mm) | 50.326 ± 5.406 | | 45.325 ± 3.758 | | 53.500 ± 4.968 | | 52.519 ± 6.004 | |
| | Co-Go (mm) | 65.215 ± 6.903 | | 60.548 ± 5.356 | | 68.604 ± 6.447 | | 67.409 ± 6.712 | |
| | Convexity (A-NPo) (mm) | -3.313 ± 2.132 | | -2.027 ± 1.233 | | -2.387 ± 1.922 | | -3.566 ± 2.178 | |
| | Maxillary Skeletal (A-Na Perp) (mm) | 1.074 ± 3.263 | | -0.723 ± 2.835 | | 0.873 ± 2.921 | | 2.226 ± 2.421 | |
| | Midface Length (Co-A) (mm) | 82.603 ± 7.088 | | 82.682 ± 4.894 | | 86.111 ± 6.248 | | 84.600 ± 5.704 | |
| | Ar - A (mm) | 81.287 ± 6.524 | | 81.323 ± 5.011 | | 85.227 ± 6.201 | | 84.351 ± 6.012 | |
| | Maxillary length (ANS-PNS) (mm) | 49.810 ± 4.588 | | 50.841 ± 2.974 | | 52.511 ± 3.567 | | 51.500 ± 3.651 | |
| | Pog - NB (mm) | 2.551 ± 2.242 | | 2.357 ± 1.694 | | 2.156 ± 1.866 | | 3.566 ± 1.796 | |
| | Mand. Skeletal (Pg-Na Perp) (mm) | 8.536 ± 7.085 | | 2.423 ± 5.825 | | 6.267 ± 5.572 | | 10.623 ± 4.812 | |
| | Mandibular Body Length (Go-Gn)(mm) | 85.674 ± 7.414 | | 81.893 ± 5.319 | | 81.388 ± 5.545 | | 85.353 ± 7.075 | |
| | Length of Mand Base (Go-Pg)(mm) | 74.274 ± 6.629 | | 71.952 ± 3.737 | | 70.483 ± 4.505 | | 73.523 ± 5.286 | |
| | Mandibular length (Co-Gn)(mm) | 123.046 ± 12.052 | | 116.475 ± 6.136 | | 121.500 ± 7.992 | | 118.332 ± 8.352 | |
| | Co-B1 Total mand (mm) | 120.644 ± 11.657 | | 114.420 ± 6.292 | | 119.287 ± 7.672 | | 115.677 ± 8.065 | |
| | Ar - Gn (mm) | 116.328 ± 10.870 | | 109.184 ± 5.831 | | 115.060 ± 7.406 | | 112.791 ± 8.438 | |
| | Basal Width (mm) | 6.451 ± 1.806 | | 7.136 ± 1.109 | | 7.462 ± 1.532 | | 7.881 ± 1.384 | |
| | Mx/Md diff (Co-Gn - Co-A)(mm) | 40.446 ± 6.836 | | 33.780 ± 3.413 | | 35.378 ± 4.181 | | 33.740 ± 4.678 | |
| | Wits (FOP) (mm) | -8.700 ± 4.657 | | -5.839 ± 2.706 | | -5.777 ± 3.061 | | -5.381 ± 2.680 | |
| | Wits Appraisal (mm) | -7.597 ± 4.176 | | -4.811 ± 2.353 | | -4.744 ± 3.008 | | -4.594 ± 2.107 | |
Each value represents the mean and standard deviation (SD).

## Slide 3
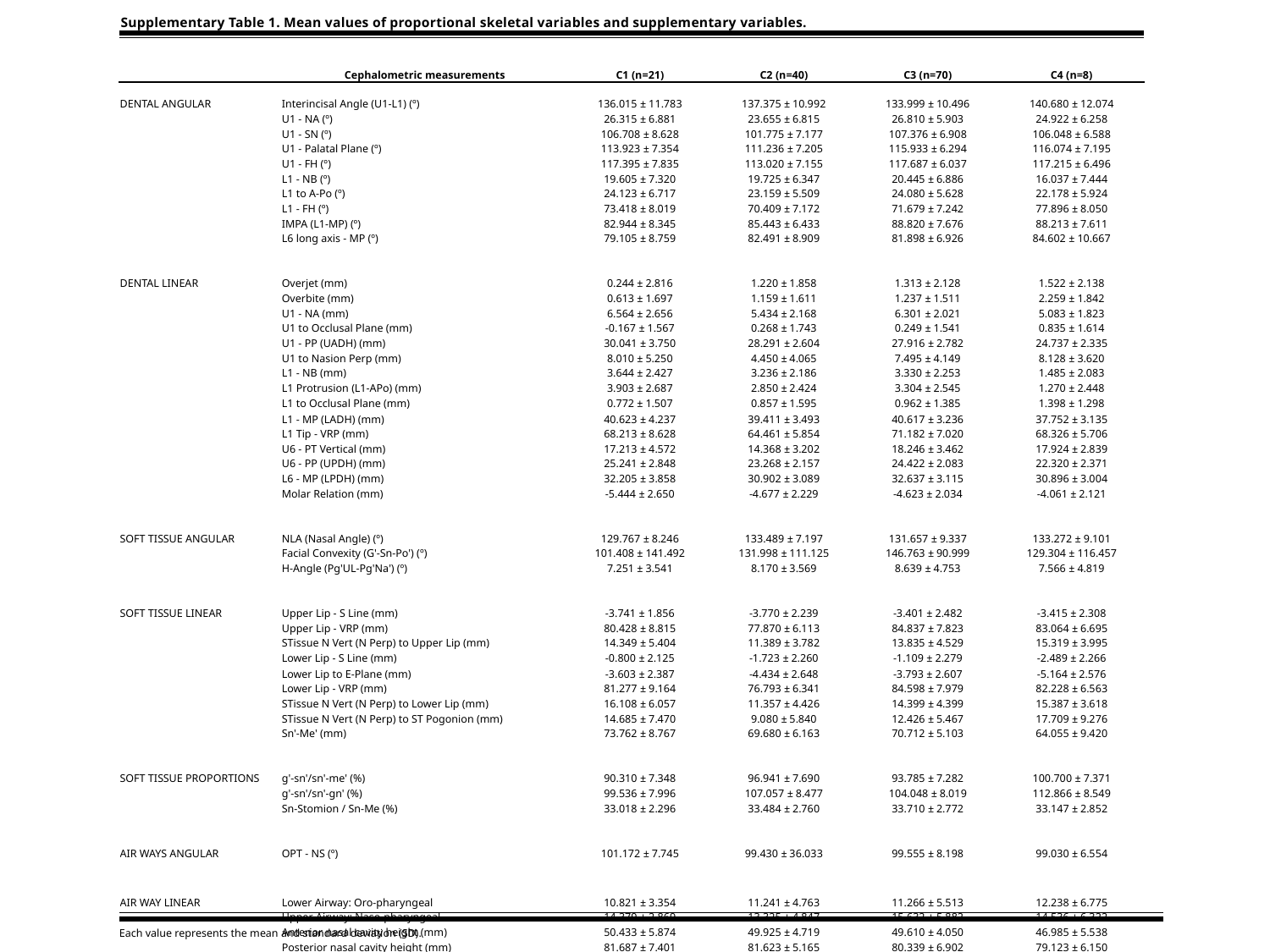

Supplementary Table 1. Mean values of proportional skeletal variables and supplementary variables.
| | Cephalometric measurements | C1 (n=21) | | C2 (n=40) | | C3 (n=70) | | C4 (n=8) | |
| --- | --- | --- | --- | --- | --- | --- | --- | --- | --- |
| DENTAL ANGULAR | Interincisal Angle (U1-L1) (º) | 136.015 ± 11.783 | | 137.375 ± 10.992 | | 133.999 ± 10.496 | | 140.680 ± 12.074 | |
| | U1 - NA (º) | 26.315 ± 6.881 | | 23.655 ± 6.815 | | 26.810 ± 5.903 | | 24.922 ± 6.258 | |
| | U1 - SN (º) | 106.708 ± 8.628 | | 101.775 ± 7.177 | | 107.376 ± 6.908 | | 106.048 ± 6.588 | |
| | U1 - Palatal Plane (º) | 113.923 ± 7.354 | | 111.236 ± 7.205 | | 115.933 ± 6.294 | | 116.074 ± 7.195 | |
| | U1 - FH (º) | 117.395 ± 7.835 | | 113.020 ± 7.155 | | 117.687 ± 6.037 | | 117.215 ± 6.496 | |
| | L1 - NB (º) | 19.605 ± 7.320 | | 19.725 ± 6.347 | | 20.445 ± 6.886 | | 16.037 ± 7.444 | |
| | L1 to A-Po (º) | 24.123 ± 6.717 | | 23.159 ± 5.509 | | 24.080 ± 5.628 | | 22.178 ± 5.924 | |
| | L1 - FH (º) | 73.418 ± 8.019 | | 70.409 ± 7.172 | | 71.679 ± 7.242 | | 77.896 ± 8.050 | |
| | IMPA (L1-MP) (º) | 82.944 ± 8.345 | | 85.443 ± 6.433 | | 88.820 ± 7.676 | | 88.213 ± 7.611 | |
| | L6 long axis - MP (º) | 79.105 ± 8.759 | | 82.491 ± 8.909 | | 81.898 ± 6.926 | | 84.602 ± 10.667 | |
| | | | | | | | | | |
| | | | | | | | | | |
| DENTAL LINEAR | Overjet (mm) | 0.244 ± 2.816 | | 1.220 ± 1.858 | | 1.313 ± 2.128 | | 1.522 ± 2.138 | |
| | Overbite (mm) | 0.613 ± 1.697 | | 1.159 ± 1.611 | | 1.237 ± 1.511 | | 2.259 ± 1.842 | |
| | U1 - NA (mm) | 6.564 ± 2.656 | | 5.434 ± 2.168 | | 6.301 ± 2.021 | | 5.083 ± 1.823 | |
| | U1 to Occlusal Plane (mm) | -0.167 ± 1.567 | | 0.268 ± 1.743 | | 0.249 ± 1.541 | | 0.835 ± 1.614 | |
| | U1 - PP (UADH) (mm) | 30.041 ± 3.750 | | 28.291 ± 2.604 | | 27.916 ± 2.782 | | 24.737 ± 2.335 | |
| | U1 to Nasion Perp (mm) | 8.010 ± 5.250 | | 4.450 ± 4.065 | | 7.495 ± 4.149 | | 8.128 ± 3.620 | |
| | L1 - NB (mm) | 3.644 ± 2.427 | | 3.236 ± 2.186 | | 3.330 ± 2.253 | | 1.485 ± 2.083 | |
| | L1 Protrusion (L1-APo) (mm) | 3.903 ± 2.687 | | 2.850 ± 2.424 | | 3.304 ± 2.545 | | 1.270 ± 2.448 | |
| | L1 to Occlusal Plane (mm) | 0.772 ± 1.507 | | 0.857 ± 1.595 | | 0.962 ± 1.385 | | 1.398 ± 1.298 | |
| | L1 - MP (LADH) (mm) | 40.623 ± 4.237 | | 39.411 ± 3.493 | | 40.617 ± 3.236 | | 37.752 ± 3.135 | |
| | L1 Tip - VRP (mm) | 68.213 ± 8.628 | | 64.461 ± 5.854 | | 71.182 ± 7.020 | | 68.326 ± 5.706 | |
| | U6 - PT Vertical (mm) | 17.213 ± 4.572 | | 14.368 ± 3.202 | | 18.246 ± 3.462 | | 17.924 ± 2.839 | |
| | U6 - PP (UPDH) (mm) | 25.241 ± 2.848 | | 23.268 ± 2.157 | | 24.422 ± 2.083 | | 22.320 ± 2.371 | |
| | L6 - MP (LPDH) (mm) | 32.205 ± 3.858 | | 30.902 ± 3.089 | | 32.637 ± 3.115 | | 30.896 ± 3.004 | |
| | Molar Relation (mm) | -5.444 ± 2.650 | | -4.677 ± 2.229 | | -4.623 ± 2.034 | | -4.061 ± 2.121 | |
| | | | | | | | | | |
| | | | | | | | | | |
| SOFT TISSUE ANGULAR | NLA (Nasal Angle) (º) | 129.767 ± 8.246 | | 133.489 ± 7.197 | | 131.657 ± 9.337 | | 133.272 ± 9.101 | |
| | Facial Convexity (G'-Sn-Po') (º) | 101.408 ± 141.492 | | 131.998 ± 111.125 | | 146.763 ± 90.999 | | 129.304 ± 116.457 | |
| | H-Angle (Pg'UL-Pg'Na') (º) | 7.251 ± 3.541 | | 8.170 ± 3.569 | | 8.639 ± 4.753 | | 7.566 ± 4.819 | |
| | | | | | | | | | |
| | | | | | | | | | |
| SOFT TISSUE LINEAR | Upper Lip - S Line (mm) | -3.741 ± 1.856 | | -3.770 ± 2.239 | | -3.401 ± 2.482 | | -3.415 ± 2.308 | |
| | Upper Lip - VRP (mm) | 80.428 ± 8.815 | | 77.870 ± 6.113 | | 84.837 ± 7.823 | | 83.064 ± 6.695 | |
| | STissue N Vert (N Perp) to Upper Lip (mm) | 14.349 ± 5.404 | | 11.389 ± 3.782 | | 13.835 ± 4.529 | | 15.319 ± 3.995 | |
| | Lower Lip - S Line (mm) | -0.800 ± 2.125 | | -1.723 ± 2.260 | | -1.109 ± 2.279 | | -2.489 ± 2.266 | |
| | Lower Lip to E-Plane (mm) | -3.603 ± 2.387 | | -4.434 ± 2.648 | | -3.793 ± 2.607 | | -5.164 ± 2.576 | |
| | Lower Lip - VRP (mm) | 81.277 ± 9.164 | | 76.793 ± 6.341 | | 84.598 ± 7.979 | | 82.228 ± 6.563 | |
| | STissue N Vert (N Perp) to Lower Lip (mm) | 16.108 ± 6.057 | | 11.357 ± 4.426 | | 14.399 ± 4.399 | | 15.387 ± 3.618 | |
| | STissue N Vert (N Perp) to ST Pogonion (mm) | 14.685 ± 7.470 | | 9.080 ± 5.840 | | 12.426 ± 5.467 | | 17.709 ± 9.276 | |
| | Sn'-Me' (mm) | 73.762 ± 8.767 | | 69.680 ± 6.163 | | 70.712 ± 5.103 | | 64.055 ± 9.420 | |
| | | | | | | | | | |
| | | | | | | | | | |
| SOFT TISSUE PROPORTIONS | g'-sn'/sn'-me' (%) | 90.310 ± 7.348 | | 96.941 ± 7.690 | | 93.785 ± 7.282 | | 100.700 ± 7.371 | |
| | g'-sn'/sn'-gn' (%) | 99.536 ± 7.996 | | 107.057 ± 8.477 | | 104.048 ± 8.019 | | 112.866 ± 8.549 | |
| | Sn-Stomion / Sn-Me (%) | 33.018 ± 2.296 | | 33.484 ± 2.760 | | 33.710 ± 2.772 | | 33.147 ± 2.852 | |
| | | | | | | | | | |
| | | | | | | | | | |
| AIR WAYS ANGULAR | OPT - NS (º) | 101.172 ± 7.745 | | 99.430 ± 36.033 | | 99.555 ± 8.198 | | 99.030 ± 6.554 | |
| | | | | | | | | | |
| | | | | | | | | | |
| AIR WAY LINEAR | Lower Airway: Oro-pharyngeal | 10.821 ± 3.354 | | 11.241 ± 4.763 | | 11.266 ± 5.513 | | 12.238 ± 6.775 | |
| | Upper Airway: Naso-pharyngeal | 14.379 ± 2.869 | | 13.325 ± 4.847 | | 15.632 ± 5.882 | | 14.536 ± 6.322 | |
| | Anterior nasal cavity height (mm) | 50.433 ± 5.874 | | 49.925 ± 4.719 | | 49.610 ± 4.050 | | 46.985 ± 5.538 | |
| | Posterior nasal cavity height (mm) | 81.687 ± 7.401 | | 81.623 ± 5.165 | | 80.339 ± 6.902 | | 79.123 ± 6.150 | |
| | H - PP (ANS-PNS) (mm) | -61.177 ± 8.927 | | -58.364 ± 7.618 | | -61.838 ± 8.405 | | -58.766 ± 8.785 | |
| | PNS to Basion (mm) | 43.323 ± 3.622 | | 42.034 ± 3.790 | | 44.417 ± 4.482 | | 44.187 ± 3.755 | |
Each value represents the mean and standard deviation (SD).
