# Supplementary material for: Novel Sub-Clustering of Class III Skeletal Malocclusion Phenotypes in a Southern European Population Based on Proportional Measurements
Source: J Clin Med. 2020 Sep 22;9(9):3048. doi: 10.3390/jcm9093048 (PMC7565379; doi:10.3390/jcm9093048)
Supplement: Supplementary file 1 [file jcm-09-03048-s001.zip › Supplementary Table 1. Mean values of proportional skeletal variables and supplementary variables.pdf]

Supplementary Table 1. Mean values of proportional skeletal variables and supplementary variables.

	Cephalometric measurements	C1 (n=39)	C2 (n=44)	C3 (n=82)	C4 (n=47)
SKELETAL PROPORTIONAL	P-A Face Height (S-Go/N-Me) (%)	64.967 ± 4.333	63.991 ± 3.755	68.888 ± 4.168	72.074 ± 4.972
	PFH:AFH (%)	53.023 ± 3.226	50.493 ± 3.156	57.398 ± 3.575	59.243 ± 3.783
	S-Ar/Ar-Go (%)	66.097 ± 5.615	77.800 ± 6.282	63.037 ± 5.484	64.364 ± 5.881
	UFH (N-ANS/(N-ANS+ANS-Me)) (%)	42.000 ± 1.372	44.095 ± 1.892	43.423 ± 1.458	46.426 ± 1.457
	LFH/TFH (ANS-Me:N-Me) (%)	58.092 ± 1.384	55.777 ± 1.915	56.618 ± 1.505	53.730 ± 1.491
	Face Ht Ratio (N-ANS/ANS-Me) (%)	0.721 ± 0.040	0.793 ± 0.069	0.766 ± 0.055	0.868 ± 0.062
	SN/GoMe (%)	93.615 ± 5.487	99.616 ± 6.655	105.483 ± 5.734	98.332 ± 5.427
	ANS-PNS/Me-Go (%)	0.695 ± 0.050	0.748 ± 0.050	0.794 ± 0.048	0.745 ± 0.054
	Articular Angle/SNB (%)	56.638 ± 3.999	54.542 ± 2.425	58.121 ± 4.033	57.608 ± 2.879
	Saddle-Sella Angle (SN-Ar)/ SNA (%)	64.884 ± 4.822	62.480 ± 4.231	64.659 ± 4.743	64.222 ± 3.157
	Occ Plane to FH/FMA (MP-FH) (%)	18.109 ± 15.424	23.919 ± 13.898	13.683 ± 18.126	-2.186 ± 35.586
	Occ Plane to SN/SN - GoGn (%)	46.947 ± 10.183	52.255 ± 8.561	47.619 ± 11.547	52.145 ± 13.341
	Cranio-Mx Base (SN-Palatal Plane)/ SN - GoGn (%)	21.503 ± 8.671	28.414 ± 8.174	30.808 ± 10.287	43.745 ± 9.634
	Anterior Cranial Base (SN) /Length of Mand Base (Go-Pg) (%)	89.338 ± 5.116	94.032 ± 5.917	99.534 ± 5.192	92.363 ± 4.603
	Maxillary length (ANS-PNS)/Anterior Cranial Base (SN) (%)	75.287 ± 4.186	75.364 ± 3.925	75.016 ± 3.563	76.000 ± 3.275
	Maxillary length (ANS-PNS)/Midface Length (Co-A) (%)	60.355 ± 3.179	61.570 ± 3.084	61.066 ± 2.843	60.913 ± 2.637
	Maxillary length (ANS-PNS)/Length of Mand Base (Go-Pg) (%)	67.182 ± 4.261	70.721 ± 3.537	74.573 ± 3.713	70.163 ± 4.062
	Midface Length (Co-A)/Mandibular length (Co-Gn) (%)	67.280 ± 3.258	70.999 ± 2.349	70.892 ± 2.699	71.560 ± 2.719
	Mandibular Body Length (Go-Gn)/Mandibular length (Co-Gn) (%)	69.735 ± 2.314	70.317 ± 2.831	67.028 ± 2.640	72.103 ± 2.291
	Ar - A/Ar - Gn (%)	70.040 ± 3.548	74.481 ± 2.315	74.086 ± 2.876	74.857 ± 2.850
	Posterior Cranial Base (S-Ar)/Posterior Face Height (SGo) (%)	41.560 ± 2.336	45.857 ± 2.058	40.871 ± 2.371	41.062 ± 2.358
	Ramus Height (Ar-Go)/Posterior Face Height (SGo) (%)	63.039 ± 2.201	59.122 ± 2.436	65.021 ± 2.430	64.000 ± 2.444
	Posterior Cranial Base (S-Ar)/Upper Face Height (N-ANS) (%)	64.144 ± 6.533	66.279 ± 5.715	64.594 ± 6.042	63.540 ± 5.473
	Ramus Height (Ar-Go)/Lower Face Height (ANS-Me) (%)	70.382 ± 5.618	67.306 ± 4.823	78.802 ± 5.915	85.832 ± 7.108
	Maxillary Skeletal (A-N Perp)/Mand. Skeletal (Pg-Na Perp) (%)	56.107 ± 205.423	36.464 ± 128.822	-54.800 ± 632.918	15.837 ± 25.755
	Convexity (A-NPg)/Pg - NB (%)	-84.825 ± 85.790	-154.333 ± 197.248	-107.003 ± 79.893	-117.408 ± 47.705

Each value represents the mean and standard deviation (SD).

Supplementary Table 1. Mean values of proportional skeletal variables and supplementary variables.

	Cephalometric measurements	C1 (n=21)	C2 (n=40)	C3 (n=70)	C4 (n=8)
SKELETAL ANGULAR	FH - SN (°)	10.695 ± 2.873	11.252 ± 2.615	10.307 ± 2.580	11.183 ± 2.226
	SNA (°)	80.387 ± 3.632	78.123 ± 2.701	80.560 ± 3.642	81.098 ± 2.176
	SNB (°)	82.323 ± 3.730	78.870 ± 2.873	81.817 ± 3.676	82.694 ± 2.641
	ANB (°)	-1.928 ± 1.804	-0.750 ± 0.847	-1.250 ± 1.467	-1.600 ± 1.602
	SND (°)	80.521 ± 3.865	77.018 ± 2.910	79.762 ± 3.507	81.074 ± 2.731
	Y-Axis (SGn-SN) (°)	67.500 ± 4.472	69.066 ± 3.132	65.574 ± 3.498	63.766 ± 2.899
	SN - GoGn (°)	32.405 ± 6.267	33.134 ± 4.757	27.679 ± 5.010	22.911 ± 4.849
	Cranio-Mx Base/SN-Palatal Plane (°)	7.218 ± 3.660	9.455 ± 3.130	8.560 ± 3.168	10.100 ± 3.169
	Occ Plane to SN (°)	15.449 ± 5.495	17.245 ± 3.436	13.307 ± 4.327	12.170 ± 4.036
	Occ Plane to FH (°)	4.764 ± 4.663	5.989 ± 3.790	2.996 ± 3.648	0.996 ± 3.462
	Facial Axis-Ricketts (NaBa-PtGn)(°)	89.141 ± 4.424	87.375 ± 3.139	91.572 ± 3.856	93.930 ± 3.525
	FMA (MP-FH) (°)	23.644 ± 5.704	24.155 ± 4.391	19.498 ± 4.256	13.968 ± 4.404
	Lower Face Height (ANS-Xi-Pm)(°)	47.085 ± 4.356	44.411 ± 3.277	43.577 ± 3.066	37.934 ± 2.683
	Facial Angle (FH-NPo) (°)	94.246 ± 3.442	91.327 ± 2.989	93.224 ± 2.822	95.787 ± 2.572
	N-A-Pg (°)	-6.459 ± 4.192	-4.175 ± 2.633	-4.888 ± 3.998	-7.721 ± 4.510
	Facial Taper (°)	62.118 ± 4.917	64.525 ± 3.423	67.278 ± 4.008	70.236 ± 3.812
	Gonial/Jaw Angle (Ar-Go-Me) (°)	124.395 ± 6.395	125.286 ± 6.356	123.789 ± 6.273	114.945 ± 5.915
	Upper Gonial Angle (Ar-Go-Na) (°)	47.633 ± 3.416	49.855 ± 3.752	50.680 ± 4.235	47.772 ± 3.970
	Lower Gonial Angle (Na-Go-Me) (°)	76.762 ± 6.097	75.423 ± 4.539	73.111 ± 4.249	67.179 ± 3.919
	Articular Angle (°)	145.677 ± 5.909	144.773 ± 5.850	141.116 ± 6.619	143.757 ± 5.522
	Saddle/Sella Angle (SN-Ar) (°)	124.259 ± 5.952	125.361 ± 5.262	124.901 ± 4.956	126.440 ± 3.812
	Superior Angle SN-AB (°)	85.087 ± 4.975	80.055 ± 3.553	83.676 ± 4.609	85.419 ± 4.864
	Rp-FH (°)	79.241 ± 4.614	78.868 ± 4.763	75.711 ± 4.688	79.023 ± 3.936
SKELETAL LINEAR	Anterior Cranial Base (SN) (mm)	66.190 ± 5.200	67.586 ± 4.490	70.045 ± 4.221	67.821 ± 4.823
	Anterior Face Height (NaMe) (mm)	123.246 ± 13.085	119.973 ± 8.109	119.452 ± 7.316	113.783 ± 8.475
	Upper Face Height (N-ANS) (mm)	51.897 ± 4.965	53.191 ± 3.786	52.133 ± 3.561	53.000 ± 4.008
	Lower Face Height (ANS-Me) (mm)	71.838 ± 8.505	67.518 ± 5.670	67.949 ± 4.613	61.215 ± 5.095
	Posterior Cranial Base (S-Ar) (mm)	33.149 ± 3.555	35.205 ± 3.505	33.668 ± 3.846	33.609 ± 3.171
	Posterior Face Height (SGo) (mm)	79.815 ± 7.844	76.748 ± 6.611	82.317 ± 7.373	82.000 ± 8.139
	Ramus Height (Ar-Go) (mm)	50.326 ± 5.406	45.325 ± 3.758	53.500 ± 4.968	52.519 ± 6.004
	Co-Go (mm)	65.215 ± 6.903	60.548 ± 5.356	68.604 ± 6.447	67.409 ± 6.712
	Convexity (A-NPo) (mm)	-3.313 ± 2.132	-2.027 ± 1.233	-2.387 ± 1.922	-3.566 ± 2.178
	Maxillary Skeletal (A-Na Perp) (mm)	1.074 ± 3.263	-0.723 ± 2.835	0.873 ± 2.921	2.226 ± 2.421
	Midface Length (Co-A) (mm)	82.603 ± 7.088	82.682 ± 4.894	86.111 ± 6.248	84.600 ± 5.704
	Ar - A (mm)	81.287 ± 6.524	81.323 ± 5.011	85.227 ± 6.201	84.351 ± 6.012
	Maxillary length (ANS-PNS) (mm)	49.810 ± 4.588	50.841 ± 2.974	52.511 ± 3.567	51.500 ± 3.651
	Pog - NB (mm)	2.551 ± 2.242	2.357 ± 1.694	2.156 ± 1.866	3.566 ± 1.796
	Mand. Skeletal (Pg-Na Perp) (mm)	8.536 ± 7.085	2.423 ± 5.825	6.267 ± 5.572	10.623 ± 4.812
	Mandibular Body Length (Go-Gn)(mm)	85.674 ± 7.414	81.893 ± 5.319	81.388 ± 5.545	85.353 ± 7.075
	Length of Mand Base (Go-Pg)(mm)	74.274 ± 6.629	71.952 ± 3.737	70.483 ± 4.505	73.523 ± 5.286
	Mandibular length (Co-Gn)(mm)	123.046 ± 12.052	116.475 ± 6.136	121.500 ± 7.992	118.332 ± 8.352
	Co-B1 Total mand (mm)	120.644 ± 11.657	114.420 ± 6.292	119.287 ± 7.672	115.677 ± 8.065
	Ar - Gn (mm)	116.328 ± 10.870	109.184 ± 5.831	115.060 ± 7.406	112.791 ± 8.438
	Basal Width (mm)	6.451 ± 1.806	7.136 ± 1.109	7.462 ± 1.532	7.881 ± 1.384
	Mx/Md diff (Co-Gn - Co-A)(mm)	40.446 ± 6.836	33.780 ± 3.413	35.378 ± 4.181	33.740 ± 4.678
	Wits (FOP) (mm)	-8.700 ± 4.657	-5.839 ± 2.706	-5.777 ± 3.061	-5.381 ± 2.680
	Wits Appraisal (mm)	-7.597 ± 4.176	-4.811 ± 2.353	-4.744 ± 3.008	-4.594 ± 2.107

Each value represents the mean and standard deviation (SD).

Supplementary Table 1. Mean values of proportional skeletal variables and supplementary variables.

	Cephalometric measurements	C1 (n=21)	C2 (n=40)	C3 (n=70)	C4 (n=8)
DENTAL ANGULAR	Interincisal Angle (U1-L1) (°)	136.015 ± 11.783	137.375 ± 10.992	133.999 ± 10.496	140.680 ± 12.074
	U1 - NA (°)	26.315 ± 6.881	23.655 ± 6.815	26.810 ± 5.903	24.922 ± 6.258
	U1 - SN (°)	106.708 ± 8.628	101.775 ± 7.177	107.376 ± 6.908	106.048 ± 6.588
	U1 - Palatal Plane (°)	113.923 ± 7.354	111.236 ± 7.205	115.933 ± 6.294	116.074 ± 7.195
	U1 - FH (°)	117.395 ± 7.835	113.020 ± 7.155	117.687 ± 6.037	117.215 ± 6.496
	L1 - NB (°)	19.605 ± 7.320	19.725 ± 6.347	20.445 ± 6.886	16.037 ± 7.444
	L1 to A-Po (°)	24.123 ± 6.717	23.159 ± 5.509	24.080 ± 5.628	22.178 ± 5.924
	L1 - FH (°)	73.418 ± 8.019	70.409 ± 7.172	71.679 ± 7.242	77.896 ± 8.050
	IMPA (L1-MP) (°)	82.944 ± 8.345	85.443 ± 6.433	88.820 ± 7.676	88.213 ± 7.611
	L6 long axis - MP (°)	79.105 ± 8.759	82.491 ± 8.909	81.898 ± 6.926	84.602 ± 10.667
DENTAL LINEAR	Overjet (mm)	0.244 ± 2.816	1.220 ± 1.858	1.313 ± 2.128	1.522 ± 2.138
	Overbite (mm)	0.613 ± 1.697	1.159 ± 1.611	1.237 ± 1.511	2.259 ± 1.842
	U1 - NA (mm)	6.564 ± 2.656	5.434 ± 2.168	6.301 ± 2.021	5.083 ± 1.823
	U1 to Occlusal Plane (mm)	-0.167 ± 1.567	0.268 ± 1.743	0.249 ± 1.541	0.835 ± 1.614
	U1 - PP (UADH) (mm)	30.041 ± 3.750	28.291 ± 2.604	27.916 ± 2.782	24.737 ± 2.335
	U1 to Nasion Perp (mm)	8.010 ± 5.250	4.450 ± 4.065	7.495 ± 4.149	8.128 ± 3.620
	L1 - NB (mm)	3.644 ± 2.427	3.236 ± 2.186	3.330 ± 2.253	1.485 ± 2.083
	L1 Protrusion (L1-APo) (mm)	3.903 ± 2.687	2.850 ± 2.424	3.304 ± 2.545	1.270 ± 2.448
	L1 to Occlusal Plane (mm)	0.772 ± 1.507	0.857 ± 1.595	0.962 ± 1.385	1.398 ± 1.298
	L1 - MP (LADH) (mm)	40.623 ± 4.237	39.411 ± 3.493	40.617 ± 3.236	37.752 ± 3.135
	L1 Tip - VRP (mm)	68.213 ± 8.628	64.461 ± 5.854	71.182 ± 7.020	68.326 ± 5.706
	U6 - PT Vertical (mm)	17.213 ± 4.572	14.368 ± 3.202	18.246 ± 3.462	17.924 ± 2.839
	U6 - PP (UPDH) (mm)	25.241 ± 2.848	23.268 ± 2.157	24.422 ± 2.083	22.320 ± 2.371
	L6 - MP (LPDH) (mm)	32.205 ± 3.858	30.902 ± 3.089	32.637 ± 3.115	30.896 ± 3.004
	Molar Relation (mm)	-5.444 ± 2.650	-4.677 ± 2.229	-4.623 ± 2.034	-4.061 ± 2.121
SOFT TISSUE ANGULAR	NLA (Nasal Angle) (°)	129.767 ± 8.246	133.489 ± 7.197	131.657 ± 9.337	133.272 ± 9.101
	Facial Convexity (G'-Sn-Po') (°)	101.408 ± 141.492	131.998 ± 111.125	146.763 ± 90.999	129.304 ± 116.457
	H-Angle (Pg'UL-Pg'Na') (°)	7.251 ± 3.541	8.170 ± 3.569	8.639 ± 4.753	7.566 ± 4.819
SOFT TISSUE LINEAR	Upper Lip - S Line (mm)	-3.741 ± 1.856	-3.770 ± 2.239	-3.401 ± 2.482	-3.415 ± 2.308
	Upper Lip - VRP (mm)	80.428 ± 8.815	77.870 ± 6.113	84.837 ± 7.823	83.064 ± 6.695
	STissue N Vert (N Perp) to Upper Lip (mm)	14.349 ± 5.404	11.389 ± 3.782	13.835 ± 4.529	15.319 ± 3.995
	Lower Lip - S Line (mm)	-0.800 ± 2.125	-1.723 ± 2.260	-1.109 ± 2.279	-2.489 ± 2.266
	Lower Lip to E-Plane (mm)	-3.603 ± 2.387	-4.434 ± 2.648	-3.793 ± 2.607	-5.164 ± 2.576
	Lower Lip - VRP (mm)	81.277 ± 9.164	76.793 ± 6.341	84.598 ± 7.979	82.228 ± 6.563
	STissue N Vert (N Perp) to Lower Lip (mm)	16.108 ± 6.057	11.357 ± 4.426	14.399 ± 4.399	15.387 ± 3.618
	STissue N Vert (N Perp) to ST Pogonion (mm)	14.685 ± 7.470	9.080 ± 5.840	12.426 ± 5.467	17.709 ± 9.276
	Sn'-Me' (mm)	73.762 ± 8.767	69.680 ± 6.163	70.712 ± 5.103	64.055 ± 9.420
SOFT TISSUE PROPORTIONS	g'-sn'/sn'-me' (%)	90.310 ± 7.348	96.941 ± 7.690	93.785 ± 7.282	100.700 ± 7.371
	g'-sn'/sn'-gn' (%)	99.536 ± 7.996	107.057 ± 8.477	104.048 ± 8.019	112.866 ± 8.549
	Sn-Stomion / Sn-Me (%)	33.018 ± 2.296	33.484 ± 2.760	33.710 ± 2.772	33.147 ± 2.852
AIR WAYS ANGULAR	OPT - NS (°)	101.172 ± 7.745	99.430 ± 36.033	99.555 ± 8.198	99.030 ± 6.554
AIR WAY LINEAR	Lower Airway: Oro-pharyngeal	10.821 ± 3.354	11.241 ± 4.763	11.266 ± 5.513	12.238 ± 6.775
	Upper Airway: Naso-pharyngeal	14.379 ± 2.869	13.325 ± 4.847	15.632 ± 5.882	14.536 ± 6.322
	Anterior nasal cavity height (mm)	50.433 ± 5.874	49.925 ± 4.719	49.610 ± 4.050	46.985 ± 5.538
	Posterior nasal cavity height (mm)	81.687 ± 7.401	81.623 ± 5.165	80.339 ± 6.902	79.123 ± 6.150
	H - PP (ANS-PNS) (mm)	-61.177 ± 8.927	-58.364 ± 7.618	-61.838 ± 8.405	-58.766 ± 8.785
	PNS to Basion (mm)	43.323 ± 3.622	42.034 ± 3.790	44.417 ± 4.482	44.187 ± 3.755

Each value represents the mean and standard deviation (SD).
