# Supplementary material for: Novel Sub-Clustering of Class III Skeletal Malocclusion Phenotypes in a Southern European Population Based on Proportional Measurements
Source: J Clin Med. 2020 Sep 22;9(9):3048. doi: 10.3390/jcm9093048 (PMC7565379; doi:10.3390/jcm9093048)
Supplement: Supplementary file 1 [file jcm-09-03048-s001.zip › Supplementary Table 2. Explanation of cephalometric measuremments..pptx]

## Slide 1
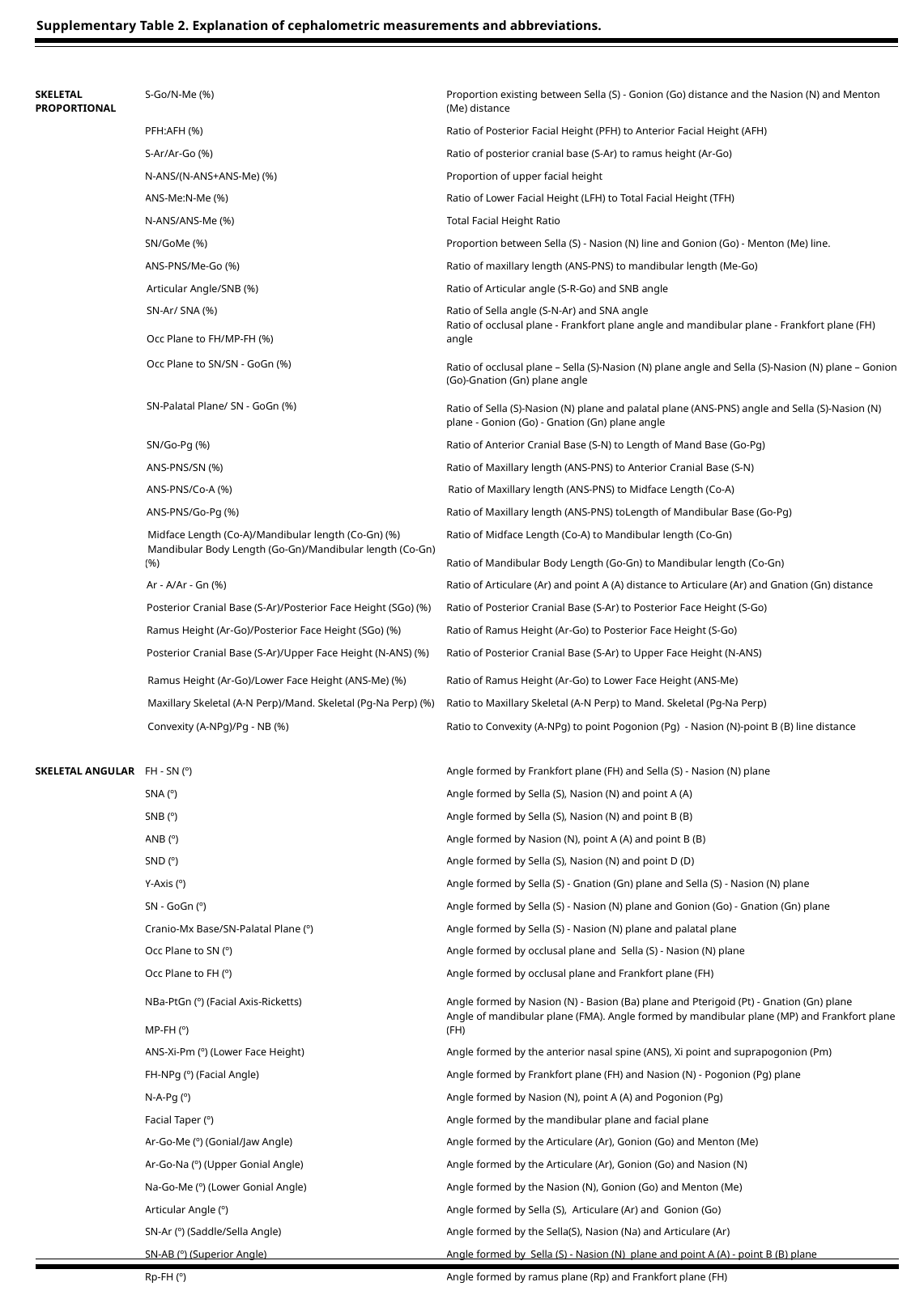

Supplementary Table 2. Explanation of cephalometric measurements and abbreviations.
| | | |
| --- | --- | --- |
| SKELETAL PROPORTIONAL | S-Go/N-Me (%) | Proportion existing between Sella (S) - Gonion (Go) distance and the Nasion (N) and Menton (Me) distance |
| | PFH:AFH (%) | Ratio of Posterior Facial Height (PFH) to Anterior Facial Height (AFH) |
| | S-Ar/Ar-Go (%) | Ratio of posterior cranial base (S-Ar) to ramus height (Ar-Go) |
| | N-ANS/(N-ANS+ANS-Me) (%) | Proportion of upper facial height |
| | ANS-Me:N-Me (%) | Ratio of Lower Facial Height (LFH) to Total Facial Height (TFH) |
| | N-ANS/ANS-Me (%) | Total Facial Height Ratio |
| | SN/GoMe (%) | Proportion between Sella (S) - Nasion (N) line and Gonion (Go) - Menton (Me) line. |
| | ANS-PNS/Me-Go (%) | Ratio of maxillary length (ANS-PNS) to mandibular length (Me-Go) |
| | Articular Angle/SNB (%) | Ratio of Articular angle (S-R-Go) and SNB angle |
| | SN-Ar/ SNA (%) | Ratio of Sella angle (S-N-Ar) and SNA angle |
| | Occ Plane to FH/MP-FH (%) | Ratio of occlusal plane - Frankfort plane angle and mandibular plane - Frankfort plane (FH) angle |
| | Occ Plane to SN/SN - GoGn (%) | Ratio of occlusal plane – Sella (S)-Nasion (N) plane angle and Sella (S)-Nasion (N) plane – Gonion (Go)-Gnation (Gn) plane angle |
| | SN-Palatal Plane/ SN - GoGn (%) | Ratio of Sella (S)-Nasion (N) plane and palatal plane (ANS-PNS) angle and Sella (S)-Nasion (N) plane - Gonion (Go) - Gnation (Gn) plane angle |
| | SN/Go-Pg (%) | Ratio of Anterior Cranial Base (S-N) to Length of Mand Base (Go-Pg) |
| | ANS-PNS/SN (%) | Ratio of Maxillary length (ANS-PNS) to Anterior Cranial Base (S-N) |
| | ANS-PNS/Co-A (%) | Ratio of Maxillary length (ANS-PNS) to Midface Length (Co-A) |
| | ANS-PNS/Go-Pg (%) | Ratio of Maxillary length (ANS-PNS) toLength of Mandibular Base (Go-Pg) |
| | Midface Length (Co-A)/Mandibular length (Co-Gn) (%) | Ratio of Midface Length (Co-A) to Mandibular length (Co-Gn) |
| | Mandibular Body Length (Go-Gn)/Mandibular length (Co-Gn) (%) | Ratio of Mandibular Body Length (Go-Gn) to Mandibular length (Co-Gn) |
| | Ar - A/Ar - Gn (%) | Ratio of Articulare (Ar) and point A (A) distance to Articulare (Ar) and Gnation (Gn) distance |
| | Posterior Cranial Base (S-Ar)/Posterior Face Height (SGo) (%) | Ratio of Posterior Cranial Base (S-Ar) to Posterior Face Height (S-Go) |
| | Ramus Height (Ar-Go)/Posterior Face Height (SGo) (%) | Ratio of Ramus Height (Ar-Go) to Posterior Face Height (S-Go) |
| | Posterior Cranial Base (S-Ar)/Upper Face Height (N-ANS) (%) | Ratio of Posterior Cranial Base (S-Ar) to Upper Face Height (N-ANS) |
| | Ramus Height (Ar-Go)/Lower Face Height (ANS-Me) (%) | Ratio of Ramus Height (Ar-Go) to Lower Face Height (ANS-Me) |
| | Maxillary Skeletal (A-N Perp)/Mand. Skeletal (Pg-Na Perp) (%) | Ratio to Maxillary Skeletal (A-N Perp) to Mand. Skeletal (Pg-Na Perp) |
| | Convexity (A-NPg)/Pg - NB (%) | Ratio to Convexity (A-NPg) to point Pogonion (Pg) - Nasion (N)-point B (B) line distance |
| | | |
| SKELETAL ANGULAR | FH - SN (º) | Angle formed by Frankfort plane (FH) and Sella (S) - Nasion (N) plane |
| | SNA (º) | Angle formed by Sella (S), Nasion (N) and point A (A) |
| | SNB (º) | Angle formed by Sella (S), Nasion (N) and point B (B) |
| | ANB (º) | Angle formed by Nasion (N), point A (A) and point B (B) |
| | SND (º) | Angle formed by Sella (S), Nasion (N) and point D (D) |
| | Y-Axis (º) | Angle formed by Sella (S) - Gnation (Gn) plane and Sella (S) - Nasion (N) plane |
| | SN - GoGn (º) | Angle formed by Sella (S) - Nasion (N) plane and Gonion (Go) - Gnation (Gn) plane |
| | Cranio-Mx Base/SN-Palatal Plane (º) | Angle formed by Sella (S) - Nasion (N) plane and palatal plane |
| | Occ Plane to SN (º) | Angle formed by occlusal plane and Sella (S) - Nasion (N) plane |
| | Occ Plane to FH (º) | Angle formed by occlusal plane and Frankfort plane (FH) |
| | NBa-PtGn (º) (Facial Axis-Ricketts) | Angle formed by Nasion (N) - Basion (Ba) plane and Pterigoid (Pt) - Gnation (Gn) plane |
| | MP-FH (º) | Angle of mandibular plane (FMA). Angle formed by mandibular plane (MP) and Frankfort plane (FH) |
| | ANS-Xi-Pm (º) (Lower Face Height) | Angle formed by the anterior nasal spine (ANS), Xi point and suprapogonion (Pm) |
| | FH-NPg (º) (Facial Angle) | Angle formed by Frankfort plane (FH) and Nasion (N) - Pogonion (Pg) plane |
| | N-A-Pg (º) | Angle formed by Nasion (N), point A (A) and Pogonion (Pg) |
| | Facial Taper (º) | Angle formed by the mandibular plane and facial plane |
| | Ar-Go-Me (º) (Gonial/Jaw Angle) | Angle formed by the Articulare (Ar), Gonion (Go) and Menton (Me) |
| | Ar-Go-Na (º) (Upper Gonial Angle) | Angle formed by the Articulare (Ar), Gonion (Go) and Nasion (N) |
| | Na-Go-Me (º) (Lower Gonial Angle) | Angle formed by the Nasion (N), Gonion (Go) and Menton (Me) |
| | Articular Angle (º) | Angle formed by Sella (S), Articulare (Ar) and Gonion (Go) |
| | SN-Ar (º) (Saddle/Sella Angle) | Angle formed by the Sella(S), Nasion (Na) and Articulare (Ar) |
| | SN-AB (º) (Superior Angle) | Angle formed by Sella (S) - Nasion (N) plane and point A (A) - point B (B) plane |
| | Rp-FH (º) | Angle formed by ramus plane (Rp) and Frankfort plane (FH) |

## Slide 2
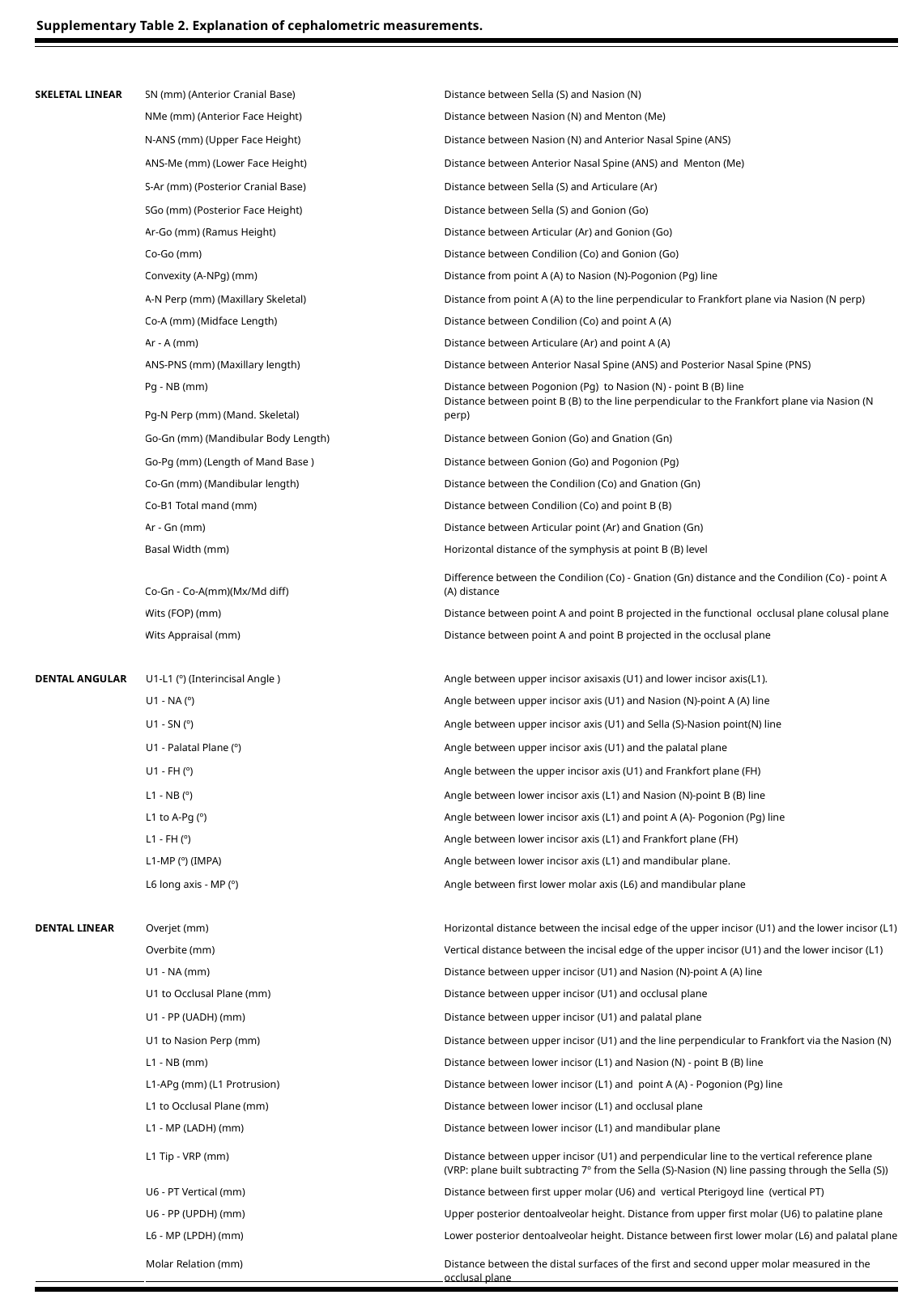

Supplementary Table 2. Explanation of cephalometric measurements.
| | | |
| --- | --- | --- |
| SKELETAL LINEAR | SN (mm) (Anterior Cranial Base) | Distance between Sella (S) and Nasion (N) |
| | NMe (mm) (Anterior Face Height) | Distance between Nasion (N) and Menton (Me) |
| | N-ANS (mm) (Upper Face Height) | Distance between Nasion (N) and Anterior Nasal Spine (ANS) |
| | ANS-Me (mm) (Lower Face Height) | Distance between Anterior Nasal Spine (ANS) and Menton (Me) |
| | S-Ar (mm) (Posterior Cranial Base) | Distance between Sella (S) and Articulare (Ar) |
| | SGo (mm) (Posterior Face Height) | Distance between Sella (S) and Gonion (Go) |
| | Ar-Go (mm) (Ramus Height) | Distance between Articular (Ar) and Gonion (Go) |
| | Co-Go (mm) | Distance between Condilion (Co) and Gonion (Go) |
| | Convexity (A-NPg) (mm) | Distance from point A (A) to Nasion (N)-Pogonion (Pg) line |
| | A-N Perp (mm) (Maxillary Skeletal) | Distance from point A (A) to the line perpendicular to Frankfort plane via Nasion (N perp) |
| | Co-A (mm) (Midface Length) | Distance between Condilion (Co) and point A (A) |
| | Ar - A (mm) | Distance between Articulare (Ar) and point A (A) |
| | ANS-PNS (mm) (Maxillary length) | Distance between Anterior Nasal Spine (ANS) and Posterior Nasal Spine (PNS) |
| | Pg - NB (mm) | Distance between Pogonion (Pg) to Nasion (N) - point B (B) line |
| | Pg-N Perp (mm) (Mand. Skeletal) | Distance between point B (B) to the line perpendicular to the Frankfort plane via Nasion (N perp) |
| | Go-Gn (mm) (Mandibular Body Length) | Distance between Gonion (Go) and Gnation (Gn) |
| | Go-Pg (mm) (Length of Mand Base ) | Distance between Gonion (Go) and Pogonion (Pg) |
| | Co-Gn (mm) (Mandibular length) | Distance between the Condilion (Co) and Gnation (Gn) |
| | Co-B1 Total mand (mm) | Distance between Condilion (Co) and point B (B) |
| | Ar - Gn (mm) | Distance between Articular point (Ar) and Gnation (Gn) |
| | Basal Width (mm) | Horizontal distance of the symphysis at point B (B) level |
| | Co-Gn - Co-A(mm)(Mx/Md diff) | Difference between the Condilion (Co) - Gnation (Gn) distance and the Condilion (Co) - point A (A) distance |
| | Wits (FOP) (mm) | Distance between point A and point B projected in the functional occlusal plane colusal plane |
| | Wits Appraisal (mm) | Distance between point A and point B projected in the occlusal plane |
| | | |
| DENTAL ANGULAR | U1-L1 (º) (Interincisal Angle ) | Angle between upper incisor axisaxis (U1) and lower incisor axis(L1). |
| | U1 - NA (º) | Angle between upper incisor axis (U1) and Nasion (N)-point A (A) line |
| | U1 - SN (º) | Angle between upper incisor axis (U1) and Sella (S)-Nasion point(N) line |
| | U1 - Palatal Plane (º) | Angle between upper incisor axis (U1) and the palatal plane |
| | U1 - FH (º) | Angle between the upper incisor axis (U1) and Frankfort plane (FH) |
| | L1 - NB (º) | Angle between lower incisor axis (L1) and Nasion (N)-point B (B) line |
| | L1 to A-Pg (º) | Angle between lower incisor axis (L1) and point A (A)- Pogonion (Pg) line |
| | L1 - FH (º) | Angle between lower incisor axis (L1) and Frankfort plane (FH) |
| | L1-MP (º) (IMPA) | Angle between lower incisor axis (L1) and mandibular plane. |
| | L6 long axis - MP (º) | Angle between first lower molar axis (L6) and mandibular plane |
| | | |
| DENTAL LINEAR | Overjet (mm) | Horizontal distance between the incisal edge of the upper incisor (U1) and the lower incisor (L1) |
| | Overbite (mm) | Vertical distance between the incisal edge of the upper incisor (U1) and the lower incisor (L1) |
| | U1 - NA (mm) | Distance between upper incisor (U1) and Nasion (N)-point A (A) line |
| | U1 to Occlusal Plane (mm) | Distance between upper incisor (U1) and occlusal plane |
| | U1 - PP (UADH) (mm) | Distance between upper incisor (U1) and palatal plane |
| | U1 to Nasion Perp (mm) | Distance between upper incisor (U1) and the line perpendicular to Frankfort via the Nasion (N) |
| | L1 - NB (mm) | Distance between lower incisor (L1) and Nasion (N) - point B (B) line |
| | L1-APg (mm) (L1 Protrusion) | Distance between lower incisor (L1) and point A (A) - Pogonion (Pg) line |
| | L1 to Occlusal Plane (mm) | Distance between lower incisor (L1) and occlusal plane |
| | L1 - MP (LADH) (mm) | Distance between lower incisor (L1) and mandibular plane |
| | L1 Tip - VRP (mm) | Distance between upper incisor (U1) and perpendicular line to the vertical reference plane (VRP: plane built subtracting 7º from the Sella (S)-Nasion (N) line passing through the Sella (S)) |
| | U6 - PT Vertical (mm) | Distance between first upper molar (U6) and vertical Pterigoyd line (vertical PT) |
| | U6 - PP (UPDH) (mm) | Upper posterior dentoalveolar height. Distance from upper first molar (U6) to palatine plane |
| | L6 - MP (LPDH) (mm) | Lower posterior dentoalveolar height. Distance between first lower molar (L6) and palatal plane |
| | Molar Relation (mm) | Distance between the distal surfaces of the first and second upper molar measured in the occlusal plane |

## Slide 3
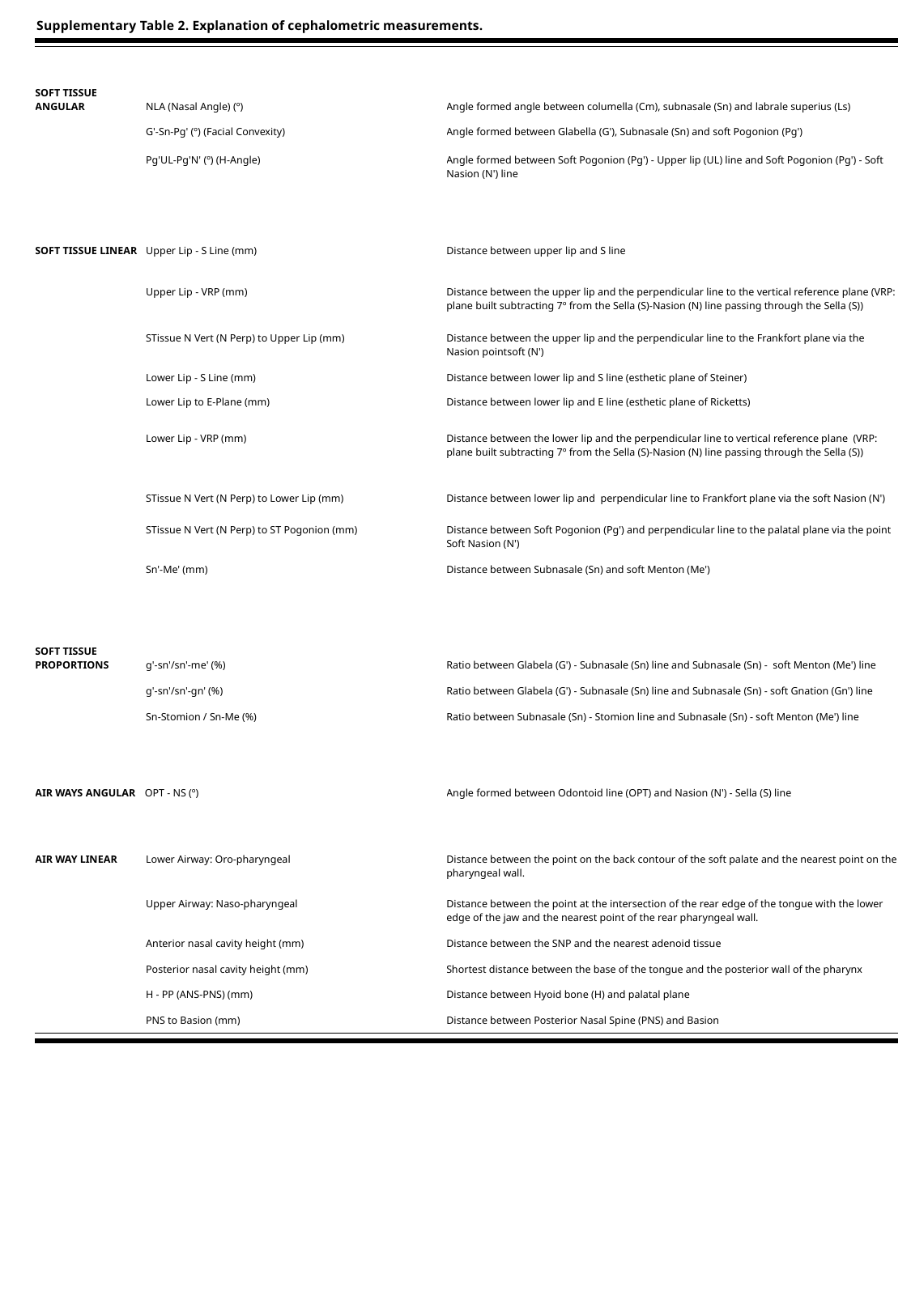

Supplementary Table 2. Explanation of cephalometric measurements.
| SOFT TISSUE ANGULAR | NLA (Nasal Angle) (º) | Angle formed angle between columella (Cm), subnasale (Sn) and labrale superius (Ls) |
| --- | --- | --- |
| | G'-Sn-Pg' (º) (Facial Convexity) | Angle formed between Glabella (G'), Subnasale (Sn) and soft Pogonion (Pg') |
| | Pg'UL-Pg'N' (º) (H-Angle) | Angle formed between Soft Pogonion (Pg') - Upper lip (UL) line and Soft Pogonion (Pg') - Soft Nasion (N') line |
| | | |
| | | |
| SOFT TISSUE LINEAR | Upper Lip - S Line (mm) | Distance between upper lip and S line |
| | Upper Lip - VRP (mm) | Distance between the upper lip and the perpendicular line to the vertical reference plane (VRP: plane built subtracting 7º from the Sella (S)-Nasion (N) line passing through the Sella (S)) |
| | STissue N Vert (N Perp) to Upper Lip (mm) | Distance between the upper lip and the perpendicular line to the Frankfort plane via the Nasion pointsoft (N') |
| | Lower Lip - S Line (mm) | Distance between lower lip and S line (esthetic plane of Steiner) |
| | Lower Lip to E-Plane (mm) | Distance between lower lip and E line (esthetic plane of Ricketts) |
| | Lower Lip - VRP (mm) | Distance between the lower lip and the perpendicular line to vertical reference plane (VRP: plane built subtracting 7º from the Sella (S)-Nasion (N) line passing through the Sella (S)) |
| | STissue N Vert (N Perp) to Lower Lip (mm) | Distance between lower lip and perpendicular line to Frankfort plane via the soft Nasion (N') |
| | STissue N Vert (N Perp) to ST Pogonion (mm) | Distance between Soft Pogonion (Pg') and perpendicular line to the palatal plane via the point Soft Nasion (N') |
| | Sn'-Me' (mm) | Distance between Subnasale (Sn) and soft Menton (Me') |
| | | |
| | | |
| SOFT TISSUE PROPORTIONS | g'-sn'/sn'-me' (%) | Ratio between Glabela (G') - Subnasale (Sn) line and Subnasale (Sn) - soft Menton (Me') line |
| | g'-sn'/sn'-gn' (%) | Ratio between Glabela (G') - Subnasale (Sn) line and Subnasale (Sn) - soft Gnation (Gn') line |
| | Sn-Stomion / Sn-Me (%) | Ratio between Subnasale (Sn) - Stomion line and Subnasale (Sn) - soft Menton (Me') line |
| | | |
| | | |
| AIR WAYS ANGULAR | OPT - NS (º) | Angle formed between Odontoid line (OPT) and Nasion (N') - Sella (S) line |
| | | |
| | | |
| AIR WAY LINEAR | Lower Airway: Oro-pharyngeal | Distance between the point on the back contour of the soft palate and the nearest point on the pharyngeal wall. |
| | Upper Airway: Naso-pharyngeal | Distance between the point at the intersection of the rear edge of the tongue with the lower edge of the jaw and the nearest point of the rear pharyngeal wall. |
| | Anterior nasal cavity height (mm) | Distance between the SNP and the nearest adenoid tissue |
| | Posterior nasal cavity height (mm) | Shortest distance between the base of the tongue and the posterior wall of the pharynx |
| | H - PP (ANS-PNS) (mm) | Distance between Hyoid bone (H) and palatal plane |
| | PNS to Basion (mm) | Distance between Posterior Nasal Spine (PNS) and Basion |
